# Potential anticancer and antioxidant lauric acid-based hydrazone synthesis and computational study toward the electronic properties†

**DOI:** 10.1039/d3ra02433d

**Published:** 2023-07-19

**Authors:** Mohammed A. Assiri, Akbar Ali, Muhammad Ibrahim, Muhammad Usman Khan, Khalid Ahmed, Muhammad Sajid Hamid Akash, Muhammad Akhtar Abbas, Athar Javed, Muhammad Suleman, Muhammad Khalid, Ishtiaq Hussain

**Affiliations:** a Chemistry Department, Faculty of Science, King Khalid University P.O. Box 9004 Abha 61413 Saudi Arabia; b Department of Chemistry, Government College University Faisalabad Faisalabad-38000 Pakistan; c Department of Applied Chemistry, Government College University Faisalabad Faisalabad-38000 Pakistan ibrahim@gcuf.edu.pk; d Department of Chemistry, University of Okara Okara-56300 Pakistan usman.chemistry@gmail.com usmankhan@uo.edu.pk; e H. E. J. Research Institute of Chemistry, International Center for Chemical and Biological Sciences, University of Karachi Karachi-75270 Pakistan; f Department of Pharaceutical Chemistry, Government College University Faisalabad Pakistan; g Department of Chemistry, Riphah International University Faisalabad Campus Pakistan; h Institute of Chemistry, Khwaja Fareed University of Engineering & Information Technology Rahim Yar Khan-64200 Pakistan; i Centre for Theoretical and Computational Research, Khwaja Fareed University of Engineering & Information Technology Rahim Yar Khan-64200 Pakistan; j Department of Pharmaceutical Sciences, Pak-Austria Fachhochschule Institute of Applied Sciences and Technology Mang Haripur Khyber Pakhtunkhwa Pakistan

## Abstract

The modification of natural products is one of the key areas of synthetic organic chemistry for obtaining valuable chemical building blocks that have medicinal significance. In this study, lauric acid-based hydrazones, namely (*E*)-*N*′-(2-nitrobenzylidene)dodecanehydrazide (NBDH), (*E*)-*N*′-(naphthalen-1-ylmethylene)dodecanehydrazide (NMDH), and (*E*)-*N*′-(4-fluorobenzylidene)dodecanehydrazide (FBDH), were synthesized and characterized using spectroscopic techniques. The newly synthesized lauric acid-based hydrazones were screened for their anticancer and antioxidant potential. The antioxidants showed their activity by inhibiting the oxidative chain reactions that produce reactive oxygen species. The antioxidant activity showed that NBDH exhibited the maximum DPPH inhibitory activity when compared with that of NMDH and FBDH, whereas the anticancer activity showed that FBDH exhibited maximum percent viability when compared to that of NBDH and NMDH. The reactivity and biological needs of the synthesized compounds NBDH, NMDH, and FBDH were met by performing geometrical, FT-IR vibrational, UV-visible, global reactivity parameters (GRP), MEP, FMO, NBO, ELF, LOL, and nonlinear optical (NLO) analysis at the DFT/B3LYP/6-311+G(d,p) level. NBO analysis confirmed the existence of extended conjugation and intramolecular charge transfer among NBDH, NMDH, and FBDH, which have the lowest gap in π → π*, which are in line with the FMO results where successful charge transfer occurred from the highest occupied molecular orbital (HOMO) to the lowest unoccupied molecular orbital (LUMO). GRP analysis confirmed the potential of NBDH, NMDH, and FBDH for biological, electronic, and NLO applications. It is clear from the comparative analysis of the urea molecule that NBDH, NMDH, and FBDH all comprise fine NLO properties.

## Abstract 2

The modification of natural products is one of the key areas of synthetic organic chemistry for obtaining valuable chemical building blocks that have medicinal significance.

## Graphical abstract

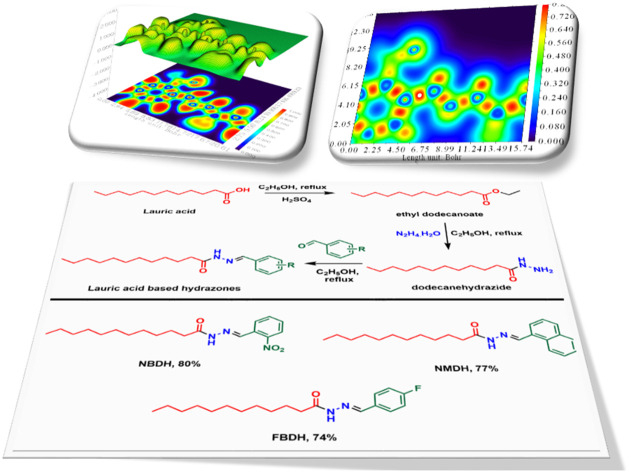

## 1 Introduction

**Fig. 1:**
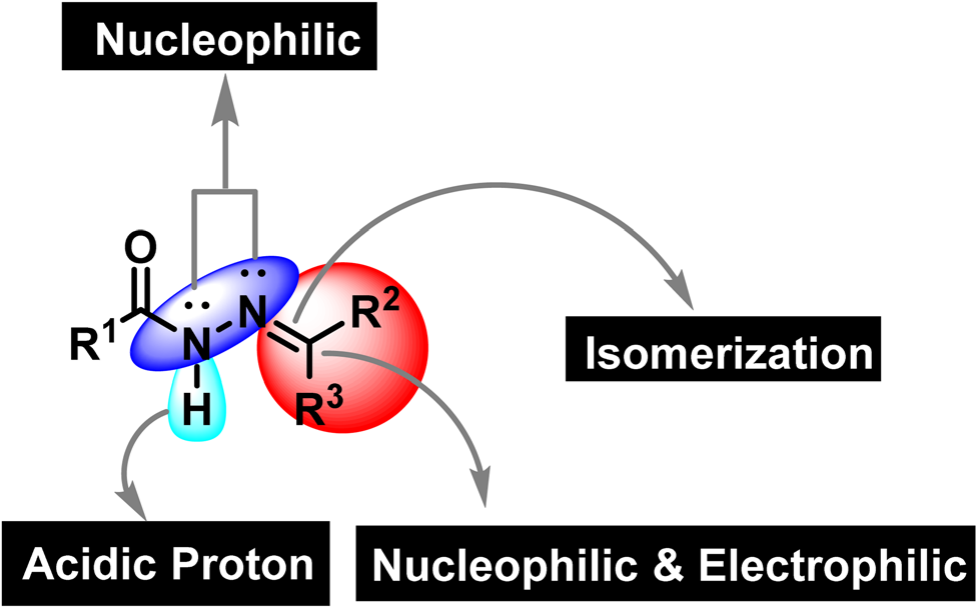
The structural assortment of hydrazone functionality.

Synthetic organic chemistry is getting recognition for the production of valuable chemical building blocks having medicinal importance. The modification of natural products is one of the key areas where new potent molecules can be produced efficiently. Cancer together with other infectious diseases are the major health concerns around the globe.^1^ According to the World Health Organization, the rate of cancer causing deaths will increase to more than double in the near future.^2^ For curing cancer, chemotherapy is one of the option that is usually employed. It is important to mention that patients are more susceptible to microbial infections after receiving cancer chemotherapy as the immune system is weakened due to the emergence of multidrug resistance.^3,4^ Hence, the synthesis of multitarget potent molecules with both anticancer and antioxidant potential are highly appreciated area for the synthetic organic community. In this scenario, intensive efforts are required to discover new anticancer molecules having resilient safety and low toxicity profiles accompanied by improved biological activity.^5^ In this regard, hydrazones are considered to be a fascinated class of organic compounds for researchers owing to their multiple medicinal applications as it has necessary features (binding cites) that are vital for medicinal applications (Fig. 1).^6^

**Fig. 2:**
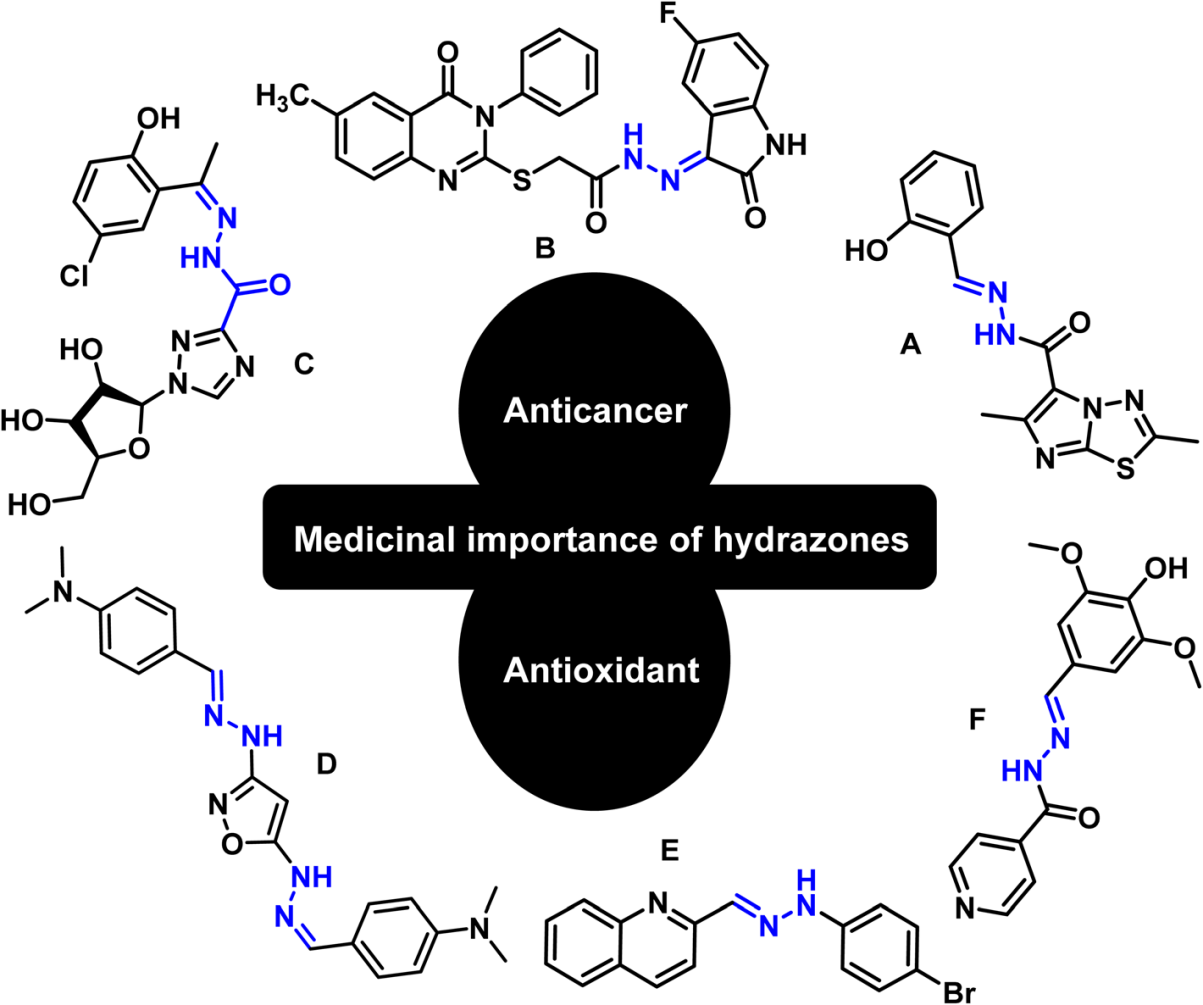
Valuable anticancer and antioxidant hydrazones from the literature.

Hydrazones are among the class of imperative compounds that have wide-ranging biological activities.^7^ Nalan Terzioglu and Aysel Gürsoy reported some new 2,6-dimethylimidazo[2,1-*b*][1,3,4]thiadiazole-5-carbohydrazide-based hydrazones, evaluated their anticancer activity, and found compound A (Fig. 2) as the most promising cytotoxic agent. The compound showed promising anticancer activity upon testing *in vitro* for National Cancer Institute's 60 human tumor cell lines.^8^ Another study also showed the anticancer potential of hydrazones by presenting an encouraging cytotoxicity for the kidney cancer cell line UO-31 (log_10_ GI_50_ value −6.68) (Fig. 2, compound B).^9^ Similarly, Wei-Yong Liu *et al.* worked on the ribavirin-based hydrazone derivatives (compound C, Fig. 2) and reported their antiproliferative activity for the A549 lung cancer cells.^10^ Beside the anticancer activity, hydrazone derivatives have shown promising antioxidant behavior. Ebraheem Abdu Musad *et al.* presented the synthesis of isoxazoles-based hydrazones that have potential antioxidant and antibacterial activities (Fig. 2, compound D).^11^ M. Orhan Puskullu and coworkers reported the synthesis of quinoline-2-carbaldehyde hydrazone derivatives and reported their antioxidant behavior (Fig. 2, compound E).^12^ Similarly, Michel Baltas and coworkers synthesized the syringic hydrazones compounds (Fig. 2, compound F) with promising antioxidant activity.^13^

The overlap of interdisciplinary studies are getting consideration to employ computational studies to the newly manufactured organic molecules to investigate their optoelectronic properties. The computational tool has been employed for freshly synthesized organic molecules for the exploration of their noncovalent interactions,^14–16^ natural bond orbitals properties,^17–19^ and nonlinear optical (NLO) behavior.^20–24^ In this scenario, herein, we are presenting our findings regarding the synthesis of lauric acid-based hydrazones, their anticancer and antioxidant behavior, as well as computational investigation.

## 2 Materials and methods

### 2.1 General procedure

Novel hydrazones have been synthesized from natural lauric acid in a stepwise manner. All the reagents used were analytical grade and solvents were used without further purification. The reaction progress was monitored by TLC cards covered with silica gel (0.25 mm thickness). The NMR spectra were recorded using a Bruker-Avance 400 MHz spectrometer, while FTIR analysis was performed using the Shimadzu IR Prestige-21.

### 2.2 Technique for the synthesis of lauric acid-based hydrazones (NBDH, NMDH, and FBDH)

A mixture of dodecanehydrazide (0.48 mmol) and substituted aromatic aldehydes (0.50 mmol) was stirred in dry ethanol, followed by 3 h reflux. After completion (monitored by TLC), the reaction mixture was concentrated using a rotary evaporator. After the workup, the final products were purified using column chromatographic techniques.

#### 2.2.1 (*E*)-*N*′-(2-Nitrobenzylidene)dodecanehydrazide (NBDH)

IR *ν*_max_ (cm^−1^) KBr: 1578 (–C=N, iminic), 1688 (–C=O, amidic), 3072 (–N–H, H-bonded); UV *λ*_max_ (nm) 245; ^1^H NMR (400 MHz, MeOD) *δ* 8.59 (s, 1H), 8.28 (d, *J* = 7.1 Hz, 1H), 8.04 (d, *J* = 4.0 Hz, 1H), 7.72 (dd, *J* = 14.0, 6.8 Hz, 2H), 2.32 (t, *J* = 7.4 Hz, 2H), 1.28 (m, 19H), 0.88 (t, *J* = 6.5 Hz, 3H). ^13^C NMR (100 MHz, MeOD) *δ* 173.26, 144.37, 140.54, 134.59, 134.24, 131.83, 129.81, 125.63, 35.53, 33.46, 33.04, 30.69, 30.58, 30.44, 30.37, 30.34, 30.26, 26.63, 26.04.

#### 2.2.2 (*E*)-*N*′-(Naphthalen-1-ylmethylene)dodecanehydrazide (NMDH)

IR *ν*_max_ (cm^−1^) KBr: 1589 (C=N, iminic), 1648 (C=O, amidic), 3072 (N–H, H-bonded); UV *λ*_max_ (nm) 285; ^1^H NMR (400 MHz, MeOD) *δ* 8.83 (s, 1H), 8.65 (d, *J* = 8.5 Hz, 1H), 8.04 (d, *J* = 7.1 Hz, 1H), 7.94–7.91 (m, 2H), 7.56–7.51 (m, 3H), 4.55 (s, 1H), 2.35 (t, *J* = 7.5 Hz, 2H), 1.75 (d, *J* = 7.8 Hz, 2H), 1.29 (s, 16H), 0.85 (d, *J* = 7.0 Hz, 3H). ^13^C NMR (100 MHz, MeOD) *δ* 173.83, 148.24, 145.53, 135.35, 132.31, 131.74, 130.71, 129.84, 128.87, 126.41, 125.17, 124.79, 35.69, 33.05, 33.02, 30.71, 30.59, 30.46, 30.41, 30.36, 30.26, 30.31, 26.79.

#### 2.2.3 (*E*)-*N*′-(4-Fluorobenzylidene)dodecanehydrazide (FBDH)

IR *ν*_max_ (cm^−1^) KBr: 1550 (–C=N, iminic), 1680 (–C=O, amidic), 3072 (–N–H, H-bonded); UV *λ*_max_ (nm) 235; ^1^H NMR (400 MHz, MeOD) *δ* 8.07 (s, 1H), 7.82 (d, *J* = 5.5 Hz, 1H), 7.70 (dd, *J* = 6.4, 3.2 Hz, 1H), 7.15 (d, *J* = 8.7 Hz, 2H), 2.29 (t, *J* = 7.5 Hz, 2H), 1.28 (s, 19H), 0.88 (t, *J* = 6.6 Hz, 3H). ^13^C NMR (100 MHz, MeOD) *δ* 172.91, 166.43, 147.49, 144.28, 130.78, 116.82, 35.56, 33.44, 33.04, 30.70, 30.58, 30.44, 30.39, 30.35, 30.28, 26.75, 26.09.

### 2.3 *In vitro* antioxidant assay

The potential antioxidant capacity of derivatized compounds (NBDH, NMDH, and FBDH) was determined by preparing the test samples in DMSO at concentrations ranging from 15.625 μg mL^−1^ to 500 μg mL^−1^. Next, 20 μL of each test sample was mixed with 180 μL of a solution containing 9.2 mg 2,2-diphenyl-1-picrylhydrazyl (DPPH) dissolved in 100 mL methanol. Ascorbic acid and DMSO were used as positive and negative controls, respectively. The reaction mixtures were incubated in the dark at 37 °C for 60 min, and the absorbance of the samples was measured at 517 nm. The percentage of inhibition or scavenging was determined by measuring the discoloration of the DPPH purple color.

### 2.4 Determination of cell viability

Human liver cancer cells (HepG2 cells) were cultured in a 96-well plate, and their viability was assessed using the MTT assay. The cells were treated with derivatized compounds (NBDH, NMDH, and FBDH) of different concentrations for 48 h, followed by the addition of MTT reagent (5 mg mL^−1^) and incubated for 4 h at 37 °C. The resulting formazan crystals were dissolved in dimethyl sulfoxide (DMSO), and the absorbance was measured at 570 nm using a microplate reader. Human liver cancer cells (HepG2 cells) were gifted by Cell and Molecular Laboratory, Department of Zoology, Government College University Faisalabad, Pakistan.

### 2.5 Determination of IC_50_ and cytotoxicity

With the help of following formula, we calculated the IC_50_ values of the three compounds.IC_50_ = (concentration of compound × 50)/% inhibition

To calculate the % inhibition, we used the following formula.% Inhibition = (control OD − (sample OD/control OD)) × 100

Similarly, with the help of the following formula, we also calculated the cytotoxicity of the three compounds.Cytotoxicy (%) = cell viability (%)

## 3 Results and discussion

**Scheme 1:**
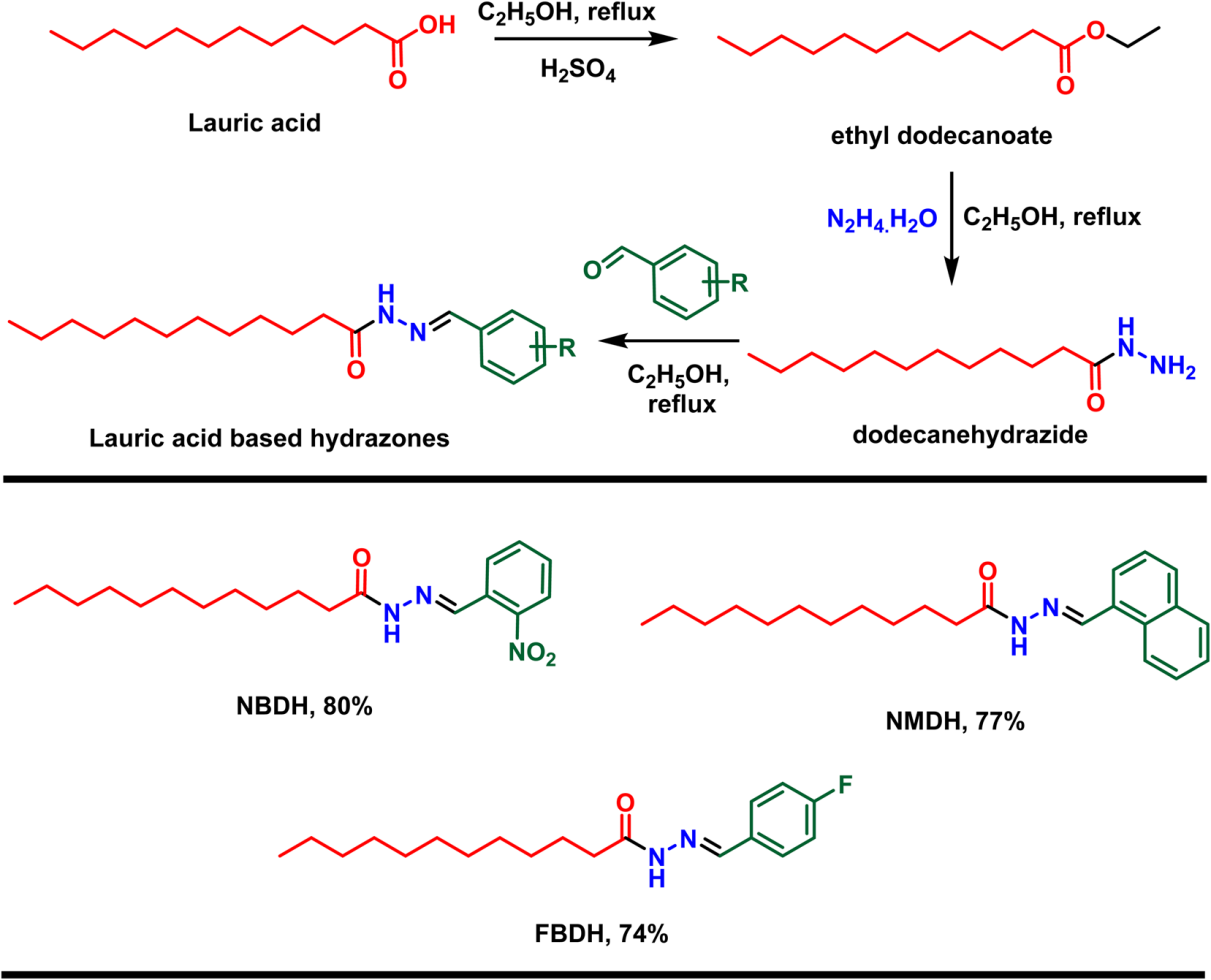
Preparation of hydrazones from natural lauric acid.

First, lauric acid was converted in to its ester using ethanol as a solvent as well as reagent in the presence of two drops of H_2_SO_4_ as catalyst under 3 h refluxing conditions. After workup and purification, the ester was reacted with hydrazine monohydrate using ethanol as the solvent, which facilitate the construction of lauric acid-based hydrazide. Finally, the hydrazide was reacted with substituted benzaldehydes in ethanol under 3 to 4 h reflux conditions to accomplished the desired hydrazones (Scheme 1).

In the current research work, lauric acid has been used as a starting material for the stepwise synthesis of hydrazones. The preparation of these hydrazones were analyzed by various spectroscopic methods. In the IR spectrum, the –OH peak of lauric acid disappeared and a signal of the aliphatic ester was observed, which supports the synthesis of targeted ethyl dodecanoate. Ethyl dodecanoate was converted to dodecanehydrazide, which is supported the by the appearance of two broad peaks at 3288 cm^−1^ and 2918 cm^−1^ of –NH stretching and disappearance of the aliphatic ester signal, respectively. Lastly, the synthesis of the targeted hydrazone was indicated by the disappearance of two peaks at 3288 cm^−1^ and 2918 cm^−1^ of –NH stretching, and new peaks emerged at 1578 (–C=N, iminic), 1689 (–C=O, amidic), and 3072 cm^−1^ (–N–H, H-bonded) in the case of (*E*)-*N*′-(2-nitrobenzylidene)dodecanehydrazide (NBDH), 1589 (C=N, iminic), 1648 (C=O, amidic), and 3072 cm^−1^ (N–H, H-bonded) in the case of (*E*)-*N*′-(naphthalen-1-ylmethylene)dodecanehydrazide (NMDH), and 1550 (–C=N, iminic), 1680 (–C=O, amidic), and 3072 cm^−1^ (–N–H, H-bonded) in the case of compound (*E*)-*N*′-(4-fluorobenzylidene)dodecanehydrazide (FBDH). In ^1^H-NMR, new peaks were observed in the aromatic area of the spectrum, which authenticated the formation of hydrazone *via* the coupling of dodecanehydrazide and substituted aromatic aldehydes.

### 3.1 Investigation of antioxidant potential of lauric acid-based hydrazones

**Fig. 3:**
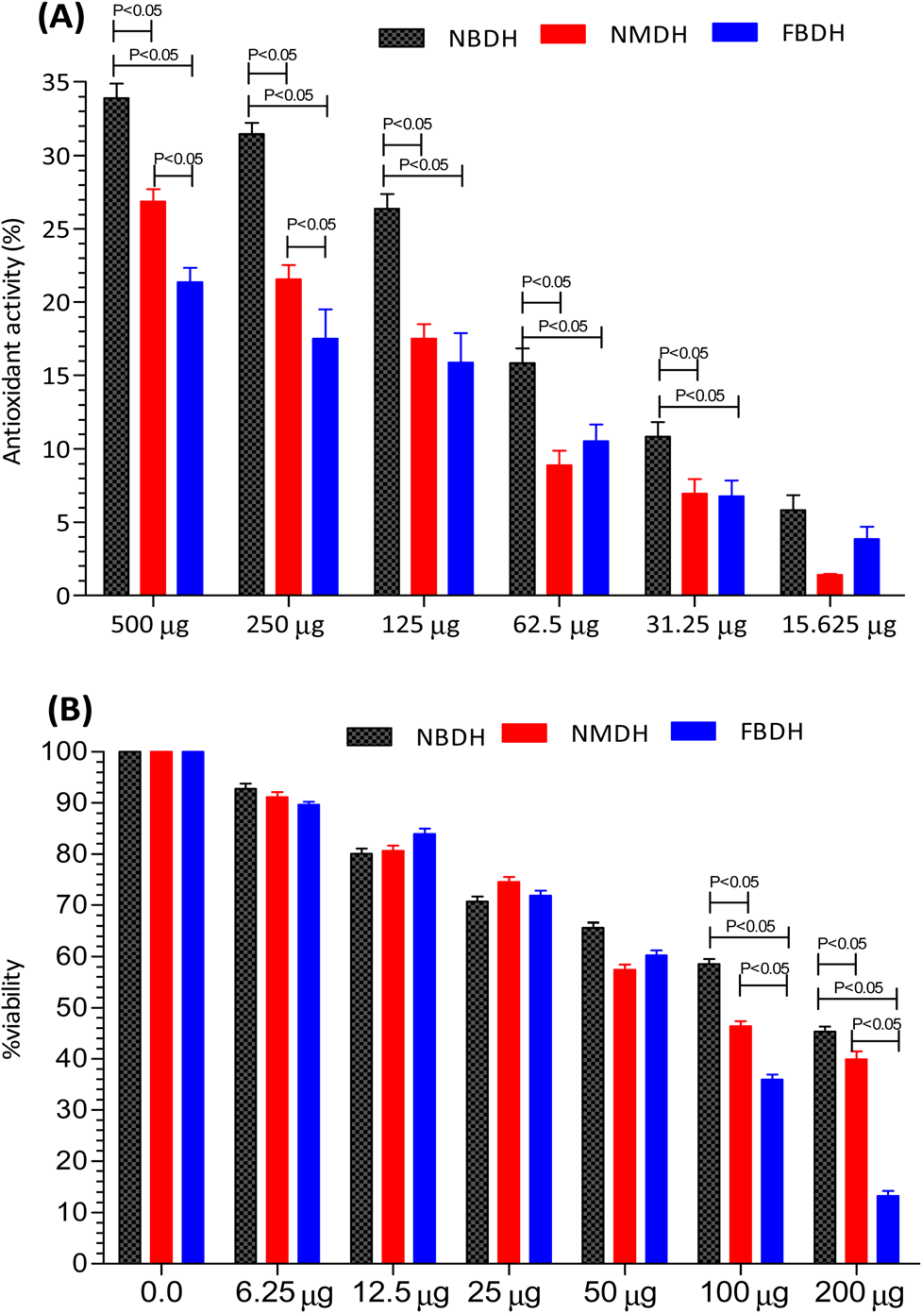
Percentage of antioxidant activity (A) and percent viability (B) of NBDH, NMDH, and FBDH in different concentrations of the extract.

The antioxidant activity of NBDH, NMDH, and FBDH was determined by DPPH assay (Fig. 3A). All the three compounds (NBDH, NMDH, and FBDH) exhibited the antioxidant potential along with an increase in their corresponding concentrations, whereas NBDH exhibited the maximum antioxidant potential (*P* < 0.05) at all concentrations when compared with the antioxidant potential of other two compounds (NMDH and FBDH) at all their corresponding concentrations. Similarly, the compound NMDH exhibited the maximum antioxidant potential at 250 μg and 500 μg when compared with that of FBDH at its same doses, respectively (Fig. 3A). However, at lower doses, a nonsignificant difference was found between NMDH and FBDH. The difference in the antioxidant potential of the derivatized compounds was due to the aromatic substitution on dodecanehydrazide. Therefore, the order of activity of the derivatized compounds against the HepG2 cells were as follows: NBDH > NMDH > FBDH.

### 3.2 Investigation of anticancer potential of lauric acid-based hydrazones

After 48 h exposure of HepG2 cells with different concentrations (6.25 μg mL^−1^ to 200 μg mL^−1^) of NBDH, NMDH, and FBDH compounds, the percent viability of HeG2 cells was observed. We observed that all the three compounds exhibited moderate to excellent antiproliferative activity, but the difference in the percent viability of the three compounds was due to the aromatic substitution on dodecanehydrazide. Compound FBDH showed maximum percent viability at 100 μg mL^−1^ and 200 μg mL^−1^ when compared with that of the other two compounds, *i.e.*, NBDH and NMDH, respectively (Fig. 3B), which showed that the compound FBDH exhibited this activity due to the presence of 4-fluorobenzylidene in its structure instead of 2-nitrobenzylidene and naphthalen-1-ylmethylene that were present in NBDH and NMDH, respectively. Therefore, the order of activity of the derivatized compounds against the HepG2 cells were as follows: FBDH > NMDH > NBDH.

### 3.3 Determination of IC_50_ and cytotoxicity

The results presented in Fig. 3B suggests that the percent cell viability results, IC_50_ values, and cytotoxicity trends are consistent. Compound FBDH exhibited the highest percent viability, indicating lower cytotoxicity and a higher IC_50_ value, while NBDH and NMDH showed progressively lower percent viability, suggesting higher cytotoxicity and lower IC_50_ values (Table 1).

**Table 1:** IC_50_ and cytotoxicity of the tested compound

Concentration of compounds	IC_50_ (μg mL^−1^)			Cytotoxicity (%)		
	NBDH	NMDH	FBDH	NBDH	NMDH	FBDH
6.25 μg	51.06 ± 11.18	47.84 ± 14.16	46.98 ± 8.74	7.188 ± 2.3	8.826 ± 2.45	10.34 ± 0.57
12.5 μg	45.03 ± 13.07	50.57 ± 3.59	53.25 ± 3.60	19.87 ± 6.54	19.33 ± 2.36	16.04 ± 2.34
25 μg	48.25 ± 6.02	52.44 ± 2.77	48.59 ± 5.73	29.26 ± 3.65	25.49 ± 3.24	28.17 ± 4.42
50 μg	57.89 ± 11.11	46.90 ± 7.4	51.13 ± 4.17	34.34 ± 6.54	42.50 ± 6.25	39.74 ± 3.56
100 μg	73.45 ± 8.51	46.96 ± 8.04	47.39 ± 6.01	41.40 ± 4.58	53.58 ± 4.56	64.01 ± 5.6
200 μg	93.83 ± 14.31	47.78 ± 2.96	44.44 ± 8.16	96.72 ± 6.58	60.05 ± 8.56	54.66 ± 3.56

### 3.4 Computational procedure

The DFT computations of NBDH, NMDH, and FBDH were carried out utilizing the Gaussian 09 (ref. 25) and the B3LYP/6-311+G(d,p) method. The optimized structures of NBDH, NMDH, and FBDH all include vibrational distribution as part of their construction. Many different types of analyses, including frontier molecular orbital (FMO) energies, natural bond orbital (NBO), molecular electrostatic potential (MEP) analysis, and NLO, use the same fundamental set of operations (DFT/B3LYP/6-311+G(d,p)). In the gas phase, UV-vis absorption tests were also carried out. Through the use of the Multiwfn 3.7 (ref. 26) tool and the multiwave analysis method, localized orbit locators (LOLs) and electron localization functions (ELFs) topological features of NBDH, NMDH, and FBDH were uncovered. The input files were organized using GaussView 5.0,^27^ PyMOLyze 2.0,^28^ Avogadro,^29^ ChemCraft,^30^ GaussSum and GaussView,^27^ and the results of the output files were also interpreted.

## 4 Results and discussion (computational)

### 4.1 Geometric parameters

**Fig. 4:**
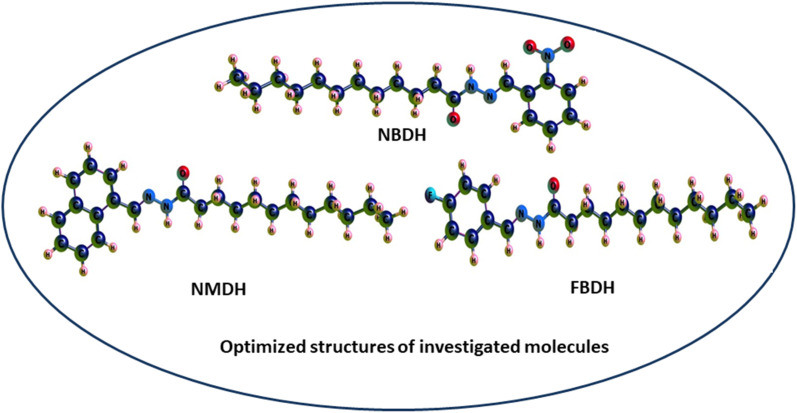
3D-sketch map of the optimized molecules.

Through a functional analysis in NBDH using the B3LYP/6-311+G(d,p) method, the optimized structural parameters (bond length and bond angles) were calculated; the results are given in Tables S1 and S2 (ESI†). The lengths of the bonds can range anywhere from 1.017 to 1.533 Å, and the bond angles can be in the range from 106.0 to 124.8°. It has been determined that C–C bonds have the longest average lengths. In a similar vein, the bonds with the greatest observed angle values are O(3)–C(2)–C(4), C(40)–C(42)–C(44), and O(53)–N(52)–O(54). DFT was used to measure the lengths of the bonds in the architecture of NBDH, and there is one benzene ring present in this architecture. The oxygen(O)–carbon(C) bond length values for C(2)–O(3) in NBDH were determined to be 1.207 Å. Benzene rings have calculated bond angles that range from 120.7° to 119.2°. The calculated bond angles between carbon, nitrogen, and oxygen for N(1)–C(2)–O(3), O(3)–C(2)–C(4), C(44)–N(52)–O(54), O(53)–N(52)–O(54), C(44)–N(52)–O(53), and O(53)–N(52)–O(54) are 122.9°, 124.0°, 118.4°, 124.1°, 117.4°, and 124.1°, respectively. The calculated bond length for C(44)–N(52), N(52)–O(53), N(52)–O(54), N(39)–C(40), N(1)–C(2), N(1)–H(15), and N(1)–N(39) are 1.480, 1.223, 1.228, 1.282, 1.395, 1.017, and 1.346 Å, respectively. The calculated bond angles between carbon, hydrogen, and nitrogen for C(2)–N(1)–H(15), C(2)–N(1)–N(39), N(1)–C(2)–C(4), H(15)–N(1)–N(39), N(1)–N(39)–C(40), N(39)–C(40)–H(41), N(39)–C(40)–C(42), C(42)–C(44)–N(52), and C(47)–C(44)–N(52) are 119.3°, 121.2°, 122.9°, 113.1°, 119.5°, 117.7°, 122.1°, 119.2°, 122.5°, and 115.4°, respectively. The bond lengths for NMDH are in the range of 1.013–1.545 Å, and the bond angles are in the range of 107.1–129.9 Å. For NMDH, DFT was used to measure the bond lengths between the two benzene rings that make up its structure. It has been determined that the bond length between C(18)–C(19), C(20)–C(21), and C(21)–C(22), respectively, has the greatest possible value. In a similar manner, the bonds that exhibit the widest possible range of angles are C(2)–C(1)–C(10), N(12)–N(13)–C(14), and N(13)–C(14)–O(15), respectively. The maximum bond length is 1.545 Å and bond angle is 129.9°. The carbon, nitrogen, and oxygen bond length values of C(11)–N(12), N(12)–N(13), N(13)–C(14), C(14)–O(15) were calculated as 1.298 Å, 1.419 Å, 1.379 Å, and 1.244 Å, respectively. The bond angles between C(10)–C(11)–N(12), N(12)–C(11)–H(34), C(11)–N(12)–N(13), N(12)–N(13)–C(14), N(12)–N(13)–H(35), C(14)–N(13)–H(35), N(13)–C(14)–O(15), N(13)–C(14)–C(16), and O(15)–C(14)–C(16) were calculated as 119.3°, 121.4°, 118.8°, 129.9°, 110.8°, 119.3°, 123.9°, 113.3°, and 122.8°, respectively. In FBDH, the bond lengths are in the range of 1.017–1.533 Å, while 106–123.5° is the range in which bond angles were calculated by DFT. The N(1)–H bond is found to have the widest possible range of bond lengths. The O(3)–O(2)–C(4) bonds are the ones that exhibit the widest range of angle possibilities. Through the use of DFT, the bond lengths that are present in the architecture of FBDH have been measured to fall somewhere in the range of 1.385–1.416 Å. There is one benzene ring that contains fluorine. The bond length values for C(2)–O(3) in FBDH is calculated through DFT as 1.209 Å. The nitrogen (N)–carbon(C) bond length values for N(39)–C(40) and N(1)–C(2) in FBDH are calculated through DFT as 1.279 Å and 1.39 Å, respectively. The calculated bond angles between carbon, nitrogen, and oxygen for C(2)–N(1)–H(15), C(2)–N(1)–N(3), N(1)–C(2)–O(3), N(1)–C(2)–C(4), H(15)–N(1)–N(39), N(1)–N(39)–C(40), and O(3)–C(2)–C(4) are 119.1°, 121.5°, 123.3°, 113.2°, 119.4°, 117.7°, and 123.5°, respectively. The DFT-obtained geometrical structures presented in Fig. 4 show that the two structural parameters agree well and can be used as a basis for further calculations.

### 4.2 FMO analysis

HOMO and LUMO are the two main orbitals that make up frontier molecular orbitals (FMOs). To describe the chemical reactivity and interaction of the molecule under study with other species, the FMOs are crucial quantum chemistry parameters. Scientists use FMOs to describe a wide range of electronic and optical characteristics, including the most reactive position in π-electron systems, reaction types in conjugated systems, and UV-vis spectra. DFT calculations at the B3LYP level of theory with 6311+G(d,p) basis set predicted the electronic properties of NBDH, NMDH, and FBDH. Table 2 shows the HOMO, LUMO, and Δ*E* values for NBDH, NMDH, and FBDH.

**Table 2:** Computed energies (*E*) and energy gap (Δ*E*) for compounds NBDH, NMDH, and FBDH^a^

MO(s)	NBDH		NMDH		FBDH	
	*E* (eV)	Δ*E* (eV)	*E* (eV)	Δ*E* (eV)	*E* (eV)	Δ*E* (eV)
HOMO	−6.66	3.87	−6.87	6.43	−7.35	7.28
LUMO	−2.79		−0.44		−0.07	
HOMO−1	−7.32	5.45	−8.06	9.00	−8.60	9.56
LUMO+1	−1.87		0.95		0.96	

a *E* = energy, Δ*E* (eV) = *E*_LUMO_ − *E*_HOMO_.

**Fig. 5:**
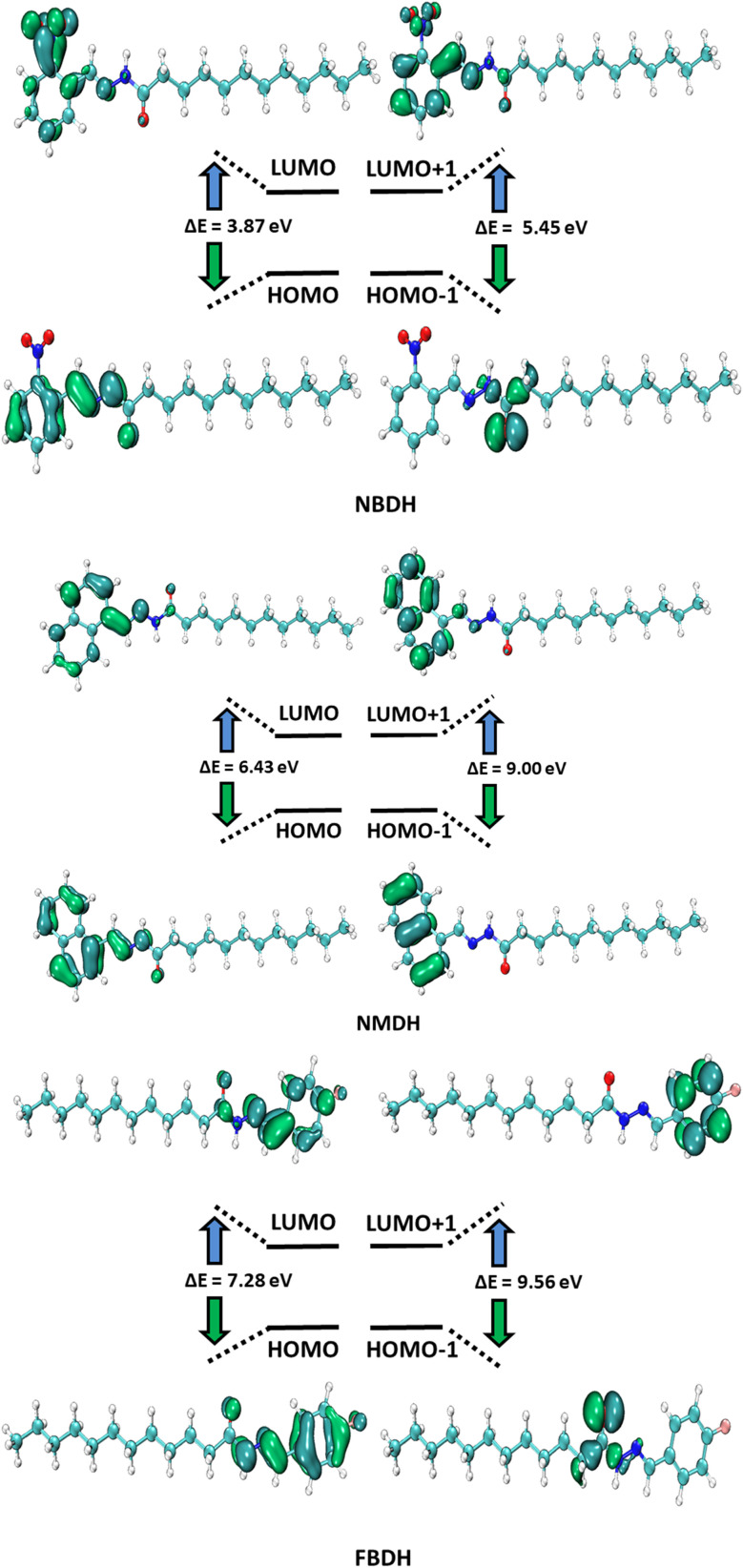
FMOs of NBDH, NMDH, and FBDH.

The pictorial displays of FMOs are presented in Fig. 5, which show how strongly deactivating group (NO_2_), slightly deactivating fluorine (F) group, and moderately activating benzene (–C_6_H_4_) substitution affect the FMOs of the investigated molecules. The value of the energy gap (Δ*E*) in FBDH was determined to be 7.28 eV, and this was found to be the highest value of band gap among all the investigated molecules. When compared with FBDH, NMDH has an energy gap that briefly decreases to 6.43 eV, which indicates the effect of slightly activating the benzene substitution in NMDH. The effect of the deactivating group can be seen quite clearly in the NBDH molecule, which demonstrates a narrowing of the energy gap to 3.87 eV, which is 3.41 eV less than that of the FBDH molecule. It is possible that the highly deactivating (NO_2_) groups are accountable for this reduction in the Δ*E* value when compared to the fluorine group. This decrease in the Δ*E* value demonstrates that the presence of highly electron-withdrawing groups brings about a decrease in the Δ*E* value. In general, the energy gap is found to decrease as one moves from FBDH to NMDH to NBDH. It is clear from this discussion that the band gap value is inversely proportional to the number of electron deactivating groups and that these groups increase as follows: –F < –C_6_H_4_ < –NO_2_. Auxiliary descriptors such as *E*_HOMO_, *E*_LUMO_, and Δ*E* are used to portray the reactivity and stability of NBDH, NMDH, and FBDH by predicting the global reactivity descriptors.

### 4.3 Global reactivity parameters (GRP)

Electron affinity (*A*) and ionization potential (*I*) were determined by means of eqn (1) and (2).

1 EA = −*E*_LUMO_

2 IP = −*E*_HOMO_

The following equations were used to calculate electronegativity, chemical hardness, chemical potential, global softness, and electrophilicity index.3
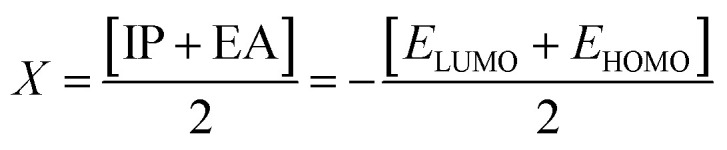
4
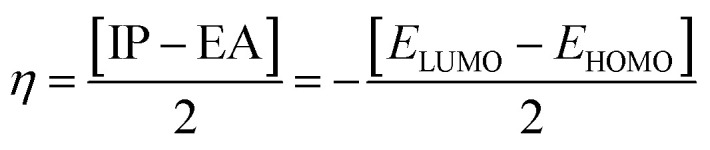
5
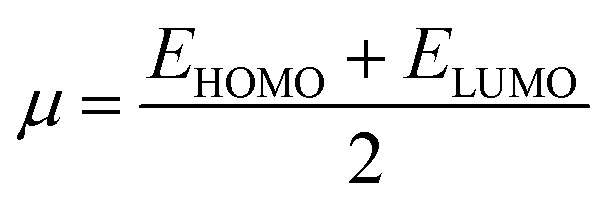
6
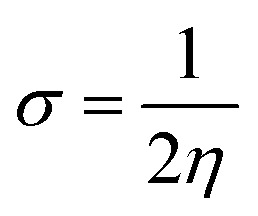


The formula for determining the electrophilicity index, denoted by the symbol (*ω*), was reported by Parr *et al.*^31–34^ as7
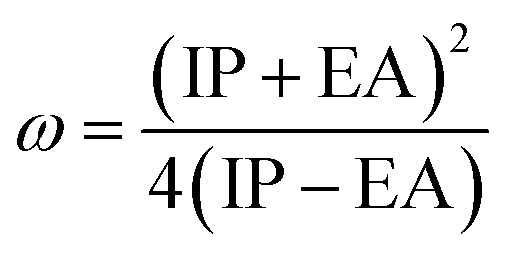


Table 3 displays the solutions to eqn (1) through (7).

**Table 3:** The global reactivity parameters of NBDH, NMDH, and FBDH

Compounds	IP	EA	*η*	*ω*	*χ*	*μ*	*σ*
NBDH	6.66	2.79	1.94	5.76	4.72	−4.72	0.26
NMDH	6.87	0.44	3.22	2.08	3.65	−3.65	0.16
FBDH	7.35	0.07	3.64	1.89	3.71	−3.71	0.14

A closer look at Table 3 reveals that FBDH has a higher donating capacity than NMDH and NBDH due to its higher overall ionization potential (IP) value of 7.35 compared to 6.87 and 6.66, respectively. NBDH has a higher EA value (2.79), indicating a greater acceptor nature, compared to NMDH (0.44), and FBDH (0.07). The positive EA values observed across the board are suggestive of the compounds' potential utility in charge-transfer reactions. FBDH is more stable and less reactive than the other compounds studied (NMDH and NBDH), as indicated by its higher global hardness (*η*) value (3.64). However, FBDH has the lowest softness (*σ*) value, leading to greater reactivity and better stability, while NBDH has the highest, indicating better reactivity and less stability. Overall, the global reactivity parameters show that FBDH has higher values for energy gap, ionization potential, and chemical hardness, which means it can donate more, is more stable, and accepts less than the other studied compounds (NBDH and NMDH). On the other hand, NBDH has higher electron affinity, electrophilicity index, and softness values than NMDH and FBDH. This means that NBDH is a compound that accepts electrons, reacts more, is less stable, and gives electrons away less than NMDH and FBDH. Also, all investigated compounds can play important roles in optoelectronic technologies.

### 4.4 DOS analysis

**Fig. 6:**
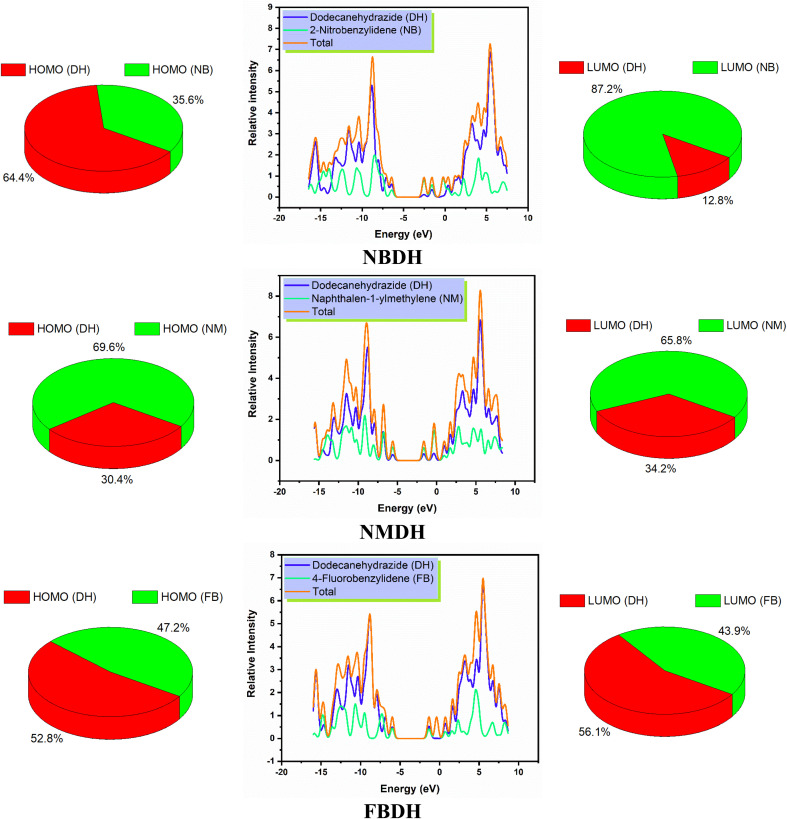
DOS spectra and percentage contribution of individual fragments of NBDH, NMDH, and FBDH.

The density of states, also known as DOS, is the ratio of distinct forms that electrons are permitted to experience at a particular energy level. It can also refer to the frequencies of electronic excitation that occur per unit of volume and unit of energy. In contrast to estimating the gap between vibrational frequencies in semiconductors, the DOS simulations make it possible to compute the Gaussian probability of various stages as a function of energy (Fig. 6).

It is necessary to have an understanding of the probability of states that are attainable per unit volume per unit of energy to measure a wide variety of electronic activities in materials with innovative designs. Some of these activities include *λ*_max_, excitation, and total electron scattering. The presence of various types of electron-activating and deactivating groups caused a shift in the distribution of electrical properties across the HOMOs and LUMOs. The electrical configuration of LUMOs was represented by positive quantities along the *x*-axis, whereas the conducting channel at HOMO was represented by negative quantities. Both of these configurations can be seen in the image below. The gap that existed between these levels was utilized as the representation for the respective energy shortfall. In the NBDH molecule, HOMO contribution originates mainly from the DH fragment (64.4%) and NB fragment (35.6%), while the in LUMO, a large contribution is given by NB (87.2%), and DH contributes about 12.8%. Due to the substitution of a slightly activating group (–C_6_H_4_) in NMDH instead of a strongly deactivating group (–NO_2_), the contribution of the DH fragment reduced to 30.4% while that of the NM fragment increases to 69.6%. The minute effect is also observed in LUMO where the DH and NM fragment contribute about 34.2% and 65.8%, respectively. The effect of a weakly deactivating group is also noted, where HOMO composition is contributed by the DH fragment (52.8%), followed by the FB fragment (47.2%). Similarly, in the LUMO, the chief contribution (56.1%) is given by DH, followed by FB is 43.9%. From this discussion, we predict that NBDH has good composition as compared to NMDH and FBDH.

### 4.5 UV-visible study

**Fig. 7:**
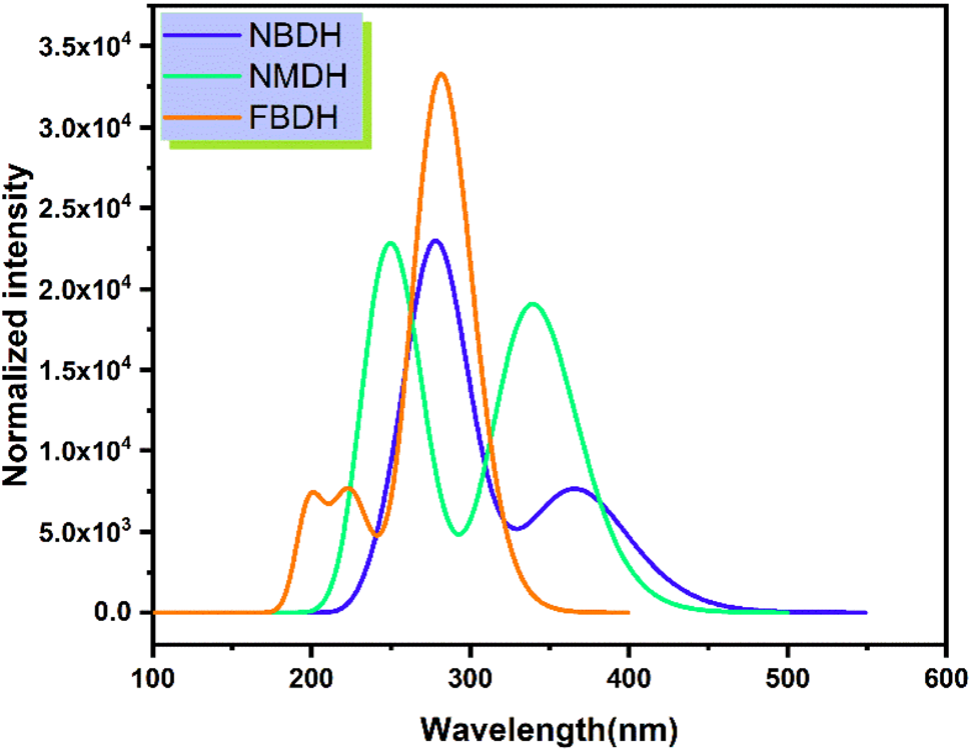
Simulated absorption spectrum of the investigated molecules.

Time-dependent DFT (TDDFT) was used at the B3LYP/6311+G(d,p) level of theory to determine the ultraviolet spectral values. The analysis of NBDH, NMDH, and FBDH using UV-visible light was completed, and the results are shown in Table 4 and Fig. 7. The wavelength of 282.94 nm and the oscillator strength of 0.42 for NBDH are both shown in Table 4; these values indicate transitions that are allowed to occur with a higher degree of freedom. Weak transitions are seen at 367.67 nm with a magnitude of 0.18 for the oscillator strength.

**Table 4:** Computed vertical excitation energies (*E*_ext_, eV), maximum wavelengths (*λ*_max_, nm), oscillator strengths (*f*_oc_), and major electronic transitions (HOMO = H, LUMO = L) of NBDH, NMDH, and FBDH

Compounds	*E*_ext_	*λ* ^DFT^_max_	*f*_oc_	Major electronic transitions
NBDH	3.37	367.67	0.18	H → L (95%)
	4.38	282.94	0.42	H−6 → L (11%), H → L+1 (73%)
NMDH	3.65	339.53	0.47	H → L (98%)
	5.11	242.42	0.35	H−1 → L (33%), H−1 → L+1 (18%), H → L+2 (39%)
FBDH	4.39	282.75	0.78	H → L (96%)
	5.52	224.67	0.17	H−2 → L (58%), H → L+1 (32%)

Table 4 shows that the wavelengths and oscillator strengths for NMDH are 339.53 nm and 242.42 eV, respectively, indicating relatively stronger allowed transitions. Weak transitions are observed at 242.42 nm with a magnitude of 0.35 for a fixed strength of the oscillator. Table 4 shows that the wavelengths for FBDH are 282.75 nm, and the oscillator strengths are 0.78, indicating that relatively stronger transitions are allowed. Weak transitions are observed at 224.67 nm and low magnitudes of oscillator strength (0.174).

### 4.6 MEP analysis

**Fig. 8:**
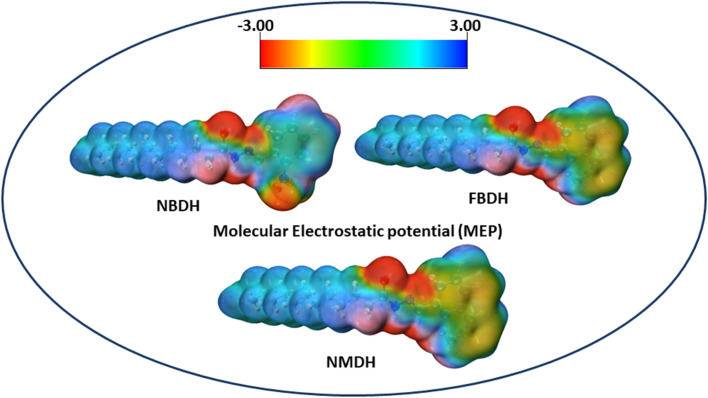
MEP analysis of NBDH, NMDH, and FBDH.

The molecular electrostatic potential is a force exerted on molecules at a given location by the molecule's electron cloud and nuclei. The most effective approach to analyze electrophilic, nucleophilic, and radical attacks is to evaluate the electrostatic potential surfaces of the molecule. Understanding macromolecules, organic molecules, and even inorganic molecules better in terms of their reactivity and biomolecular properties could be very useful. Multiwfn 3.8 was used to analyze the MEP surface, with red representing electrophilic attacks, yellow representing nucleophilic attacks, green representing radical attacks, and blue representing neutral attacks (in the range from −3.00 to +3.00 kcal mol^−1^). Electrophilic reactive sites are shown in red, and nucleophilic reactive sites are shown in blue (Fig. 8).

The MEP analysis of the molecule showed that the electron density was higher in sites with negative potential around the oxygen and nitrogen atoms. The areas of the molecule that have a positive potential are located in close proximity to the C–H groups that have a lower electron density. The green part of the graph reveals a marginally negative potential. In NBDH, the red region encompasses O3, O53, and O54 as well as N1, N39, and N52. When it comes to NMDH and FBDH, there is a red region present on O3, N1, and N39. The benzene ring and the F are located in the green region, whereas the blue region contains the C–H atoms.

### 4.7 NBO analysis

NBO analysis gives a consistent picture of the conjugative and charge transfer interactions between the electron donor and acceptor, which are important for understanding the inter and intramolecular bonding. For the purpose of transferring the electron density from occupied electron orbitals to empty ones, NBO analysis can be quite helpful.^35,36^ Assuming a second-order perturbation method, we get the stabilization energy formula, given by eqn (8).8
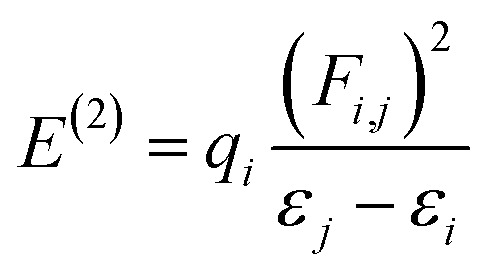
Here, *q*_*i*_ stands for the donor orbital occupancy, *F*(*i*,*j*) denotes the diagonal, and *j* and *i* are the off-diagonal NBO Fock matrix elements. The significant magnitude of the perturbation stabilization energy value, denoted by the symbol *E*^(2)^, hints at the existence of a more robust association between bonding and antibonding orbitals. NBO research on NBDH has been carried out with the B3LYP/6-311+G(d,p) level of theory, and the findings of some representative interactions have been compiled in Table S3 (ESI†).

With respective stabilization energy values of 23.69 and 5.60 kJ mol^−1^, the π(C42–C44) → π*(N52–O53) and σ(C44–C47) → σ*(C42–C44) transitions are the most likely to occur in NBDH. Conjugation in the NBDH molecule is strongly suggested by the existence of interactions (π → π*). It has been determined that the stabilization energies for the following transitions in NBDH are 7.14, 13.58, 19.86, and 21.68 kJ mol^−1^ for π(N52–O53) → π*(N52–O53), π(C47–C48) → π*(C42–C44), π(C42–C44) → π*(N39–C40), and π(C43–C45) → π*(C42–C44), respectively. Resonance transitions such as LP(2)O53 → σ*(C44–N52) and LP(3)O54 → π*(N52–O53) resulted in stabilization energy values of 13.73 and 160.86 kJ mol^−1^, respectively. The most likely transitions that take place in NMDH are π(C7–C8) → π*(C9–C10) and σ(C14–C16) → σ*(N12–N14), with respective stabilization energy values of 19.09 and 5.26 kJ mol^−1^. Both these transitions have a high probability of occurring. It was discovered that the stabilization energies in NMDH are 7.57 kJ mol^−1^ and 6.06 kJ mol^−1^ for the transitions π(C11–N12) → π*(C9–C10) and σ(C16–H37) → σ*(C14–O15), respectively. In the case of resonance, the transitions such as π(C11–N12) → π*(C9–C10) and σ(C16–H37) → σ*(C14–O15) led to stabilization energy values of 11.40, and 25.24 kJ mol^−1^, respectively. FBDH's most likely transitions are π(C43–C45) → π*(C47–C49) with a stabilization energy of 20.94 kJ mol^−1^. In resonance, the transitions LP(1)N39 → σ*(C40–C41), LP(1)N1 → π*(C2–O3), and LP(2)O3 → (N1–C2) have stabilization energy values of 10.13, 47.17, and 28.81 kJ mol^−1^. However, transitions such as σ(C49–F52) → σ*(C43–C45) and σ(C49–F52) → σ*(C44–C47) have energies of 1.35 and 1.4 kJ mol^−1^, respectively. Conjugation in the FBDH molecules aids intramolecular charge transfer, as shown in Table S3 (ESI†).

The highest probable transitions that take place in FBDH are π(C43–C45) → π*(C47–C49), having stabilization energy value of 20.94 kJ mol^−1^. In case of resonance, transitions such as LP(1)N39 → σ*(C40–C41), LP(1)N1 → π*(C2–O3), and LP(2)O3 → (N1–C2) led to the stabilization energy values 10.13, 47.17, and 28.81 kJ mol^−1^, respectively. In contrast, transitions such as σ(C49–F52) → σ*(C43–C45) and σ(C49–F52) → σ*(C44–C47) have 1.35 and 1.4 kJ mol^−1^ energies, respectively. Finally, it can be deduced on the basis of findings that conjugation is present in FBDH molecules, which ease intramolecular charge transfer. On the basis of the findings, it is possible to draw the conclusion that conjugation is present in the title molecules, which makes the process of charge transfer within molecules easier. Therefore, the powerful intramolecular conjugation present in NBDH, NMDH, and FBDH are the fundamental reasons that contribute to this system's increased degree of stability.

### 4.7 Localized orbit locator (LOL) and electron localization function (ELF) analysis

**Fig. 9:**
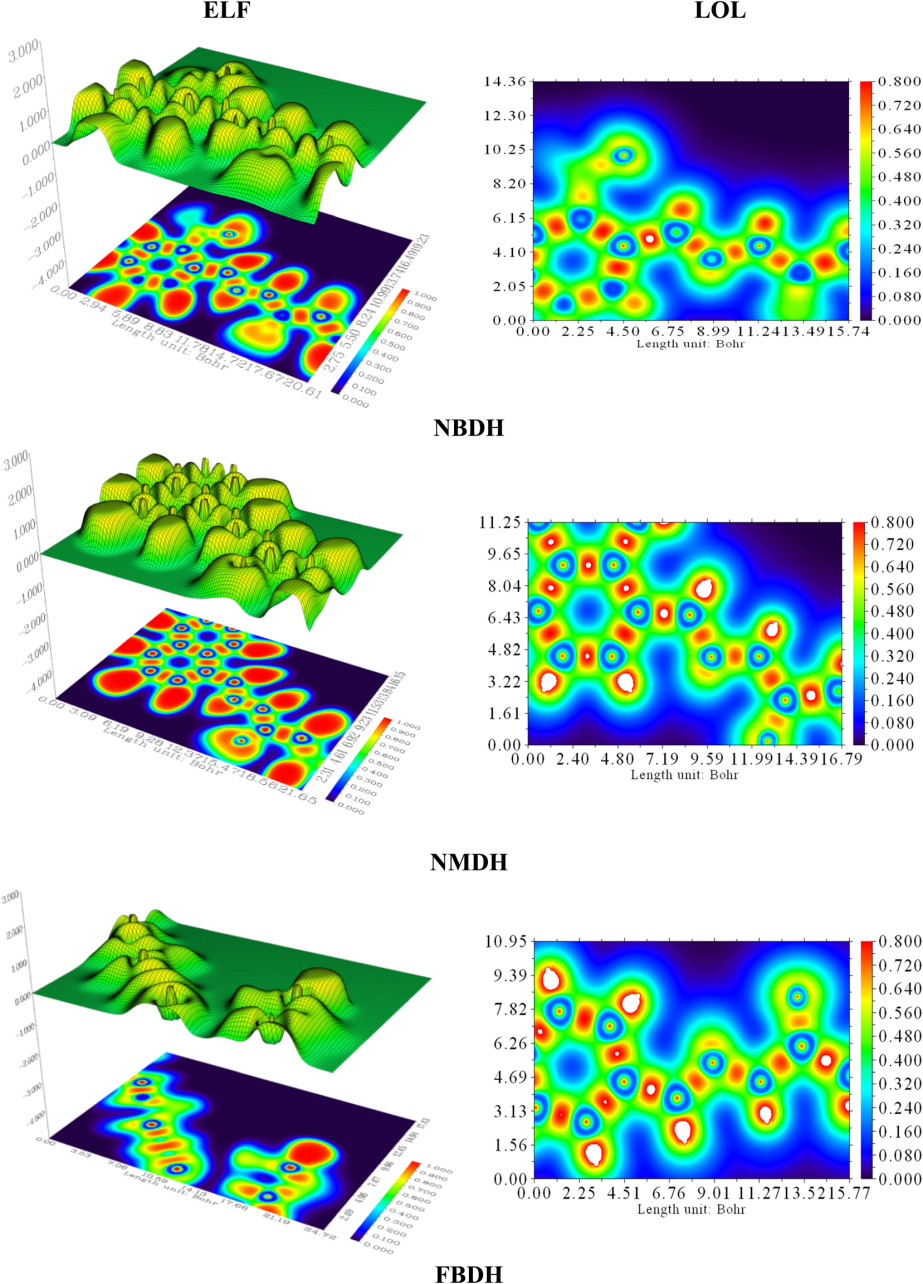
ELF and LOL analysis of NBDH, NMDH, and FBDH.

Electron localization functions (ELFs) and localized orbit locators (LOLs) were used extensively to illustrate the connections between molecular geometry and electronic properties and to facilitate a deeper understanding of the bonding dynamics of molecules' primary active sites. Using the critical points of the density function, the localized orbital locator (LOL) places emphasis on topological parameters such as the kinetic energy density. For this research, we used the computer program Multiwfn to calculate them. In this case, as shown in Fig. 9, the color red stands for areas with high LOL and ELF values and shows where bonding and nonbonding electrons are, while the blue color stands for areas with low LOL and ELF values. Both ELF and LOL have values that vary from zero. From 0 to 1.0, the range of where electrons are located is shown by values above 0.5, with a value of 0.8.

### 4.8 FT-IR analysis

**Fig. 10:**
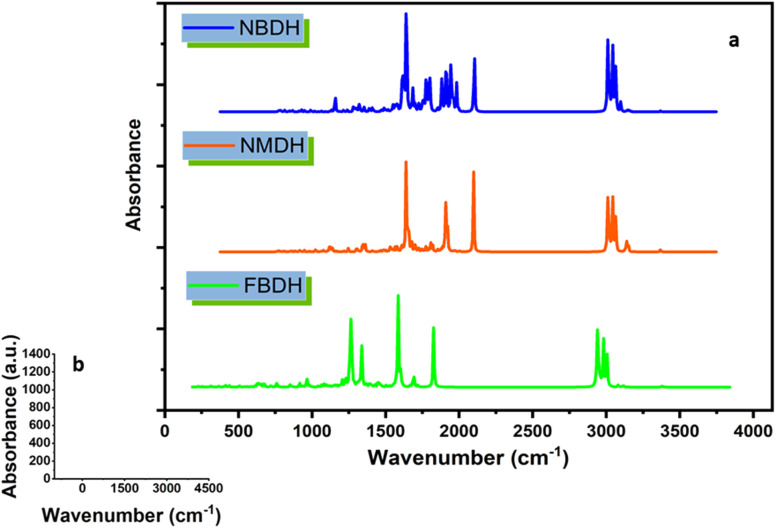
(a) IR spectra of NBDH, NMDH, and FBDH molecules obtained at the DFT/B3LYP/6-311G(d,p) level of theory, (b) shows scale along the *x* and *y* axes.

Using theoretical methods, we have completed a comprehensive vibrational analysis of the compounds of interest (NBDH, NMDH, and FBDH). The theoretical spectrum is recorded at the DFT/B3LYP/6-311G(d,p) level of theory, and the results are shown in Fig. 10. The significant unscaled and scaled^37–39^ vibrational frequencies of NBDH, NMDH, and FBDH with proposed assignments are placed in Tables S4–S6 (ESI†).

### 4.9 C–H vibrations

The majority of C–H vibrational bands are observed due to twisting, wagging, scissoring, and rocking modes of vibration, as revealed by a comparison of DFT-computed and experimentally-calculated vibrational bands. In the benzene unit of the compound NBDH, wagging mode vibrations are observed at a wavenumber of 762.53 cm^−1^. The benzene unit wavenumbers computed at 908.23 cm^−1^ correspond to twisting vibrations. The scissoring stretching modes are responsible for the vibrational band that can be seen at 708.11 cm^−1^. The calculated C–H vibrational bands were detected in NBDH at a frequency of 3127.52 cm^−1^. In the benzene unit, computed C–H symmetric stretching vibration can be found at 3224.67 and 3208.35 cm^−1^, whereas at 3193.76 cm^−1^, both asymmetric and symmetric stretching vibration can be found in the C–H of the benzene unit. At a frequency of 732.61 cm^−1^, NMDH exhibits rocking vibrations that were computed using DFT. There is evidence of a wagging mode of vibration at a frequency of 562.42 cm^−1^. At a frequency of 3049.85 cm^−1^, stretching C–H vibrational bands were detected. In the benzene unit, the computed C–H symmetric stretching vibration can be found at 3199.97 and 3198.75 cm^−1^, and the C–H asymmetric stretching vibration can be found at 3184.94 and 3163.01 cm^−1^. However, FBDH assigned the DFT frequencies of 942.11 and 827.11 cm^−1^ in the benzene unit to twisting and wagging vibrations, respectively. The calculated C–H vibrational bands in FBDH were found at 1026 cm^−1^. The C–H symmetric vibrational bands were measured at 3205.95 cm^−1^, and the C–H asymmetric vibrational bands were measured at 3193.01 cm^−1^, both in agreement with DFT calculations Tables S4–S6 (ESI†).

### 4.10 C–C stretching vibration

The vibrational modes associated with C–C stretching can typically be found in the frequency range of 1650–1400 cm^−1^. According to the findings of this research, C–C stretching vibrations are most prevalent in benzene rings. The C–C vibrational bands in the benzene ring were observed to have stretching frequencies at 1028.15, 804.57, and 651.23 cm^−1^ in FBDH, 661.65, 654.61, 562.42, and 546.69 cm^−1^ in the extended conjugating benzene unit of NMDH, and 853.63, 708.11, and 649.87 cm^−1^ in NBDH (Tables S4–S6 (ESI†)).

### 4.11 N–O vibrations

It is only possible in NBDH to observe the vibrational mode of N–O scissoring, which has stretching frequencies of 853.3 and 708.11 cm^−1^, respectively.

### 4.12 C–F vibrations

C–F vibrations are seen at 804.57 cm^−1^ in the studied molecule FBDH. This is mostly because of the deactivating group attached to the benzene unit.

### 4.13 Methyl group vibration

The asymmetric vibrations of the methyl group in compound NBDH were calculated using DFT and were found to occur at 3084.10 and 3079.35 cm^−1^. Asymmetric vibrations, as calculated by DFT, are also present at 3083.93, 3084.01, and 3079.30 cm^−1^ in NMDH and FBDH, respectively. Both NMDH and FBDH exhibit symmetric vibrations at 3018.39 cm^−1^ and a wagging mode of vibration at 896.77 cm^−1^ (Tables S4–S6 (ESI†)).

### 4.14 Ethyl group vibration

Vibrations in the ethyl group are most often seen as rocking, scissor, and twisting bands, as calculated by density functional theory. The C–H of the ethyl group in the NBDH compound exhibits asymmetric vibrations with wavenumbers of 3082.00, 3079.35, and 3041.59 cm^−1^. Twisting vibrations are given a wavenumber of 881.28 cm^−1^. The stretching rocking modes account for the vibratory band detected at 826.85 cm^−1^. DFT calculations show that vibrations at 622.50 cm^−1^ are present in NMDH, indicating the presence of rocking motions. The frequencies of 3081.83 and 3024.17 cm^−1^ are associated with asymmetric and symmetric vibrations, respectively. Asymmetric C–H vibrations are accounted for by the DFT frequencies of 3081.85, 3079.30, 3055.12, and 3041.88 cm^−1^ calculated for FBDH. Bands of vibrational energy are detected at 938.42 and 880.68 cm^−1^ because of the twisting and scissoring stretching modes, respectively. The ethyl group exhibits a rocking mode of vibrations with wavenumbers of 732.25 and 634.17 cm^−1^ (Tables S4–S6 (ESI†)).

### 4.15 N–H band vibration

The stretching frequencies of 3489.05, 3486.67, and 3491.28 cm^−1^ were observed to be associated with vibrational bands that are caused by N–H in NBDH, NMDH, and FBDH.

### 4.16 Nonlinear optical effects

Nonlinear optical (NLO) properties are exhibited by organic materials used in optical interconnections. We use DFT to draw conclusions about the NLO activities of NBDH, NMDH, and FBDH. Urea is the molecule that is most frequently used when conducting research on the NLO properties of the compound. The results of the calculations for the significant NLO parameters of NBDH, NMDH, and FBDH are presented in Tables 5 and 6. According to the findings, the maximum value of the dipole moment observed in FBDH is calculated to be 4.7054 D. On the other hand, out of all the molecules that were looked into, the value of NBDH's computed dipole moment was the smallest at 0.7839 D. The values of the computed dipole moment are found to be in the order FBDH > NMDH > NBDH, with NBDH being at the bottom of the list. The total dipole moment of the urea molecule is 1.3732 D, and the urea molecule is considered to be the standard molecule for comparing the results of dipole moment and *β*_tot_. Calculations show that the dipole moments of NBDH, NMDH, and FBDH are 0.589, 2.766, and 3.332 times greater than those of the urea molecule, respectively. Table 5 contains a tabulation of the findings regarding the major polarizability tensors of the *x*, *y*, and *z*-directions as well as the total linear polarizability (*α*). According to the findings, the value of the dipole polarizability tensors of FBDH is discovered to be greater in all the three directions when compared to the values of the polarizability tensors of the other compounds that were investigated. The computed value of dipole polarizability is found to be the highest in NMDH at 308.48 (a.u.), while the value of dipole polarizability is observed to be the lowest in FBDH at 256.55 (a.u.). NMDH > NBDH > FBDH is the observed decreasing order of computed dipole polarizability observed in the compounds that were investigated. Because of their extended conjugation, NMDH molecules have the highest dipole polarizability. Table 6 contains a listing of the values for the total first hyperpolarizability (also known as the polarizability of the second order or the NLO of the second order; *β*_tot_) as well as its contributing tensors. While the dominant transitions 1488.73 and 298.55 (a.u.) can be seen along the *x*-axis with a positive direction in NBDH and FBDH, the dominant transitions 134.15 (a.u.) can be seen along the *x*-axis with a negative direction in NMDH. In NBDH and NMDH, the highest and lowest values for tot that could be computed were 1587.43 (a.u.) and 131.02 (a.u.), respectively. NBDH > FBDH > NMDH is the decreasing order of the total observed in the molecules that have been studied. The fundamental structures of NBDH conceal the secret that explains why it has a higher NLO activity level compared to other compounds that have been investigated. In comparison to NMDH, which has a benzene group that only has a very slight activating effect, NBDH has highly deactivating groups (–NO_2_), which results in an increase in resonance as well as charge transfer. In addition, FBDH has a greater NLO response in comparison to NMDH because of the slightly deactivating group (F).

**Table 5:** Dipole moment (Debye), dipole polarizabilities, and major contributing tensors (a.u.) of NBDH, NMDH, and FBDH

Molecules	*μ*	*α*_*xx*_	*α*_*yy*_	*α*_*zz*_	〈*α*〉
NBDH	0.7839	419.99	241.80	161.00	274.26
NMDH	4.13873	469.37	286.35	169.72	308.48
FBDH	4.7054	401.39	213.06	155.21	256.55

**Table 6:** The computed second-order polarizabilities (*β*_tot_) and major contributing tensors (a.u.) of NBDH, NMDH, and FBDH

Molecules	*β*_*XXX*_	*β*_*XYY*_	*β*_*XZZ*_	*β*_*YYY*_	*β*_*XXY*_	*β*_*YZZ*_	*β*_*ZZZ*_	*β*_*XXZ*_	*β*_*YYZ*_	*β*_total_
NBDH	1488.73	34.97	64.86	355.73	116.49	19.26	−18.62	−0.90	−26.84	1587.43
NMDH	−134.15	−63.78	0.50	127.55	−6.31	−8.12	25.67	7.96	33.40	131.02
FBDH	298.55	92.87	56.74	143.26	34.22	8.44	−27.80	36.92	24.72	486.38

## 5 Conclusion

Natural products modification for the search of new chemical architectures with potential medicinal importance is one of the key area of synthetic organic chemistry. In the current research, natural lauric acid have been synthetically modified to hydrazones (*E*)-*N*′-(2-nitrobenzylidene)dodecanehydrazide (NBDH), (*E*)-*N*′-(naphthalen-1-ylmethylene)dodecanehydrazide (NMDH), and (*E*)-*N*′-(4-fluorobenzylidene)dodecanehydrazide (FBDH) in a stepwise manner and were characterized using spectroscopic techniques. The anticancer and antioxidant potential of these hydrazones have been tested. Accordingly, antioxidant agents showed that their antioxidant activity against oxidative stress by inhibiting the oxidative chain reactions that produced reactive oxygen species, and this can be accomplished by inhibiting or limiting the supply of nutrients (such as proteins and lipids) that are necessary for catalyzing these chain reactions. Anticancer agents showed that their anticancer activity in inhibiting the progression of cancer by different pathways such as inhibiting the cell division by cell cycle arrest, apoptosis by intrinsic and extrinsic pathway, and alteration in the expression of genes involved in cell proliferation. In addition, the novel hydrazones NBDH, NMDH, and FBDH were synthesized and characterized using experimental as well as computational FT-IR vibrational, UV-visible, global reactivity parameters (GRP), MEP, FMO, NBO, ELF, LOL, and nonlinear optical (NLO) analysis. The energy gap order NBDH (3.87 eV) < NMDH (6.43 eV) < FBDH (7.28 eV) is found to be inversely proportional to the number of electron deactivating groups as follows: –F < –C_6_H_4_ < –NO_2_. DOS analysis showed that HOMO contribution in NBDH, NMDH, and FBDH originates mainly from DH (64.4%), NM (69.6%), and DH (52.8%) fragments and LUMO from NB (87.2%), NM (65.8%), and DH (56.1%) respectively. NBO results confirmed that the hyperconjugative interactions among the bonds due to the delocalization of electrons on whole systems provide stability to the investigated compounds. Global reactivity results indicate that NBDH is a soft compound that accepts electrons, reacts more, is less stable, and gives electrons away less than NMDH and FBDH due to their larger energy gap. GR parameters, MEP surfaces, and ELF, LOL analysis reveal that the molecule might be bioactive. The first hyper polarizability is found to be 1654.10, 229.06, and 486.38 (a.u.) for NBDH, NMDH, and FBDH, respectively. The greater value of NBDH as compared to the other investigated molecule may be due to the strongly deactivating group effect. Findings show the evidence of promising NLO performance and may have potential NLO applications.

## Conflicts of interest

There are no conflicts to declare.

## Supplementary Material

RA-013-D3RA02433D-s001
