# Supplementary material for: Potential anticancer and antioxidant lauric acid-based hydrazone synthesis and computational study toward the electronic properties
Source: RSC Adv. 2023 Jul 19;13(31):21793–807. doi: 10.1039/d3ra02433d (PMC10354594; doi:10.1039/d3ra02433d)
Supplement: RA-013-D3RA02433D-s001 [file RA-013-D3RA02433D-s001.pdf]

Electronic Supplementary Material (ESI) for RSC Advances.
This journal is © The Royal Society of Chemistry 2023

Supplementary Information

Potential anticancer and antioxidant lauric acid based hydrazones synthesis and computational slant towards the electronic properties

Mohammed A. Assiri,[1] Akbar Ali,[2] Muhammad Ibrahim,*[3] Muhammad Usman Khan,*[4] Khalid Ahmed,[5] Muhammad Sajid Hamid Akash,[6] Akhtar Abbas,[3] Athar Javed,[3] Muhammad Suleman,[7] Muhammad Khalid,[8,9] Ishtiaq Hussain,[10]

[1]Department of Chemistry, Faculty of Science, King Khalid University, P.O. Box 9004, Abha 61413, Saudi Arabia

[2]Department of Chemistry, Government College University Faisalabad, Faisalabad-38000, Pakistan

[3]Department of Applied Chemistry, Government College University Faisalabad, Faisalabad-38000, Pakistan

[4]Department of Chemistry, University of Okara, Okara-56300, Pakistan

[5]H. E. J. Research Institute of Chemistry, International Center for Chemical and Biological Sciences, University of Karachi, Karachi-75270, Pakistan

[6]Department of Pharaceutical Chemistry, Government College University Faisalabad

[7]Department of Chemistry, Riphah International University Faisalabad Campus. Pakistan

[8]Institute of Chemistry, Khwaja Fareed University of Engineering & Information Technology, Rahim Yar Khan-64200, Pakistan

[9]Centre for Theoretical and Computational Research, Khwaja Fareed University of Engineering & Information Technology, Rahim Yar Khan-64200, Pakistan

[10]Department of Pharmaceutical Sciences, Pak-Austria fachhachole Institute of applied sciences and technology Mang Haripure, Khyber Pakhtunkhwa, Pakistan

*** Corresponding authors E-mail addresses:**

Corresponding author[1]: ibrahim@gcuf.edu.pk

Corresponding author[2]: usman.chemistry@gmail.com ; usmankhan@uo.edu.pk

Table S1: Bond length (Å) in optimized in NBDH, NMDH, and FBDH structures

NBDH		NMDH		FBDH	
**Bond Length (Å)**		**Bond Length (Å)**		**Bond Length (Å)**	
N(1)-C(2)	1.395	C(1)-C(6)	1.439	N(1)-C(2)	1.39
N(1)-H(15)	1.017	C(1)-C(10)	1.439	N(1)-H(15)	1.017
N(1)-N(39)	1.346	C(2)-C(3)	1.377	N(1)-N(39)	1.354
C(2)-O(3)	1.207	C(2)-H(27)	1.082	C(2)-O(3)	1.209
C(2)-C(4)	1.525	C(3)-C(4)	1.416	C(2)-C(4)	1.526
C(4)-C(5)	1.532	C(3)-H(28)	1.084	C(4)-C(5)	1.532
C(4)-H(16)	1.096	C(4)-C(5)	1.374	C(4)-H(16)	1.096
C(4)-H(17)	1.098	C(4)-H(29)	1.084	C(4)-H(17)	1.098
C(5)-C(6)	1.532	C(5)-C(6)	1.423	C(5)-C(6)	1.532
C(5)-H(18)	1.096	C(5)-H(30)	1.085	C(5)-H(18)	1.096
C(5)-H(19)	1.093	C(6)-C(7)	1.42	C(5)-H(19)	1.093
C(6)-C(7)	1.533	C(7)-C(8)	1.378	C(6)-C(7)	1.533
C(6)-H(20)	1.098	C(7)-H(31)	1.085	C(6)-H(20)	1.098
C(6)-H(21)	1.098	C(8)-C(9)	1.407	C(6)-H(21)	1.098
C(7)-C(8)	1.533	C(8)-H(32)	1.084	C(7)-C(8)	1.533
C(7)-H(22)	1.097	C(9)-C(10)	1.388	C(7)-H(22)	1.098
C(7)-H(23)	1.097	C(9)-H(33)	1.083	C(7)-H(23)	1.097
C(8)-C(9)	1.533	C(10)-C(11)	1.469	C(8)-C(9)	1.533
C(8)-H(24)	1.098	C(11)-N(12)	1.298	C(8)-H(24)	1.098
C(8)-H(25)	1.098	C(11)-H(34)	1.079	C(8)-H(25)	1.098
C(9)-C(10)	1.533	N(12)-N(13)	1.419	C(9)-C(10)	1.533
C(9)-H(26)	1.098	N(13)-C(14)	1.379	C(9)-H(26)	1.098
C(9)-H(27)	1.098	N(13)-H(35)	1.013	C(9)-H(27)	1.098
C(10)-C(11)	1.533	C(14)-O(15)	1.244	C(10)-C(11)	1.533
C(10)-H(28)	1.098	C(14)-C(16)	1.534	C(10)-H(28)	1.098
C(10)-H(29)	1.098	C(16)-C(17)	1.54	C(10)-H(29)	1.098
C(11)-C(12)	1.533	C(16)-H(36)	1.097	C(11)-C(12)	1.533
C(11)-H(30)	1.098	C(16)-H(37)	1.1	C(11)-H((30)	1.098
C(11)-H(31)	1.098	C(17)-C(18)	1.542	C(11)-H(31)	1.098
C(12)-C(13)	1.533	C(17)-H(38)	1.097	C(12)-C(13)	1.533
C(12)-H(32)	1.098	C(17)-H(39)	1.095	C(12)-H((32)	1.098
C(12)-H(33)	1.098	C(18)-C(19)	1.545	C(12)-H(33)	1.098
C(13)-C(14)	1.531	C(18)-H(40)	1.099	C(13)-C(14)	1.531
C(13)-H(34)	1.097	C(18)-H(41)	1.099	C(13)-H(34)	1.097
C(13)-H(35)	1.097	C(19)-C(20)	1.544	C(13)-H(35)	1.097
C(14)-H(36)	1.095	C(19)-H(42)	1.099	C(14)-H(36)	1.095
C(14)-H(37)	1.095	C(19)-H(43)	1.099	C(14)-H(37)	1.095
C(14)-H(38)	1.093	C(20)-C(21)	1.545	C(14)-H(38)	1.093
N(39)-C(40)	1.282	C(20)-H(44)	1.099	N(39)-C(40)	1.279
C(40)-H(41)	1.088	C(20)-H(45)	1.099	C(40)-H(41)	1.099
C(40)-C(42)	1.471	C(21)-C(22)	1.545	C(40)-C(42)	1.464
C(42)-C(43)	1.408	C(21)-H(46)	1.099	C(42)-C(43)	1.405
C(42)-C(44)	1.41	C(21)-H(47)	1.099	C(42)-C(44)	1.401
C(43)-C(45)	1.385	C(22)-C(23)	1.545	C(43)-C(45)	1.387
C(43)-H(46)	1.082	C(22)-H(48)	1.099	C(43)-H(46)	1.083
C(44)-C(47)	1.395	C(22)-H(49)	1.099	C(44)-C(47)	1.392
C(44)-N(52)	1.48	C(23)-C(24)	1.544	C(44)-H(48)	1.086
C(45)-C(48)	1.397	C(23)-H(50)	1.099	C(45)-C(49)	1.397

C(45)-H(49)	1.084	C(23)-H(51)	1.099	C(45)-H((50))	1.084
C(47)-C(48)	1.385	C(24)-C(25)	1.545	C(47)-C(49)	1.392
C(47)-H(50)	1.081	C(24)-H(52)	1.099	C(47)-H(51)	1.084
C(48)-H(51)	1.083	C(24)-H(53)	1.099	C(49)-H(52)	1.084
N(52)-O(53)	1.223	C(25)-C(26)	1.544		
N(52)-O(54)	1.228	C(25)- H (54)	1.098		
		C(25)-H(55)	1.098		
		C(26)-H(56)	1.096		
		C(26)-H(57)	1.096		

Table S2: Bond angle (°) in optimized in NBDH, NMDH, and FBDH structures.

NBDH		NMDH		FBDH	
**Bond angle (°)**		**Bond angle (°)**		**Bond angle (°)**	
C(2)-N(1)-H(15)	119.3	C(2)-C(1)-C(6)	118	C(2)-N(1)-H(15)	119.1
C(2)-N(1)-N(39)	121.2	C(2)-C(1)-C(10)	123.6	C(2)-N(1)-N(39)	121.5
N(1)-C(2)-O(3)	122.9	C(1)-C(2)-C(3)	121.1	N(1)-C(2)-O(3)	123.3
N(1)-C(2)-C(4)	113.1	C(1)-C(2)-H(27)	119.8	N(1)-C(2)-C(4)	113.2
H(15)-N(1)-N(39)	119.5	C(6)-C(1)-C(10)	118.4	H(15)-N(1)-N(39)	119.4
N(1)-N(39)-C(40)	117.7	C(1)-C(6)-C(5)	119.3	N(1)-N(39)-C(40)	117.7
O(3)-C(2)-C(4)	124	C(1)-C(6)-C(7)	119.6	O(3)-C(2)-C(4)	123.5
C(2)-C(4)-C(5)	112.5	C(1)-C(10)-C(9)	119.6	C(2)-C(4)-C(5)	112.3
C(2)-C(4)-H(16)	110.6	C(1)-C(10)-C(11)	120.6	C(2)-C(4)-H(16)	111
C(2)-C(4)-H(17)	106.8	C(3)-C(2)-H(27)	119	C(2)-C(4)-H(17)	106.8
C(5)-C(4)-H(16)	110.6	C(2)-C(3)-C(4)	120.7	C(5)-C(4)-H(16)	110.6
C(5)-C(4)-H(17)	109.7	C(2)-C(3)-H(28)	119.8	C(5)-C(4)-H(17)	109.5
C(4)-C(5)-C(6)	113	C(4)-C(3)-H(28)	119.5	C(4)-C(5)-C(6)	113.1
C(4)-5(H)-18(H)	109.1	C(3)-C(4)-C(5)	119.9	C(4)-C(5)-H(18)	109.1
C(4)-C(5)-H(19)	108.4	C(3)-C(4)-H(29)	119.7	C(4)-C(5)-H(19)	108.2
H(16)-C(4)-H(17)	106.3	C(5)-C(4)-H(29)	120.4	H(16)-C(4)-H(17)	106.5
C(6)-C(5)-H(18)	109.7	C(4)-C(5)-C(6)	121	C(6)-C(5)-H(18)	109.7
C(6)-C(5)-H(19)	110.3	C(4)-C(5)-H(30)	120.6	C(6)-C(5)-H(19)	110.3
(C(5)-C(6)-C(7)	113.2	C(6)-C(5)-H(30)	118.4	C(5)-C(6)-C(7)	113.2
C(5)-C(6)-H(20)	109.7	C(5)-C(6)-C(7)	121.1	C(5)-C(6)-H(20)	109.7
C(5)-C(6)-H(21)	109.3	C(6)-C(7)-C(8)	120.7	C(5)-C(6)-H(21)	109.2
H(18)-C(5)-H(19)	106.1	C(6)-C(7)-H(31)	118.6	H(18)-C(5)-H(19)	106.2
C(7)-C(6)-H(20)	109.2	C(8)-C(7)-H(31)	120.7	C(7)-C(6)-H(20)	109.2
C(7)-C(6)-H(21)	109.2	C(7)-C(8)-C(9)	120.2	C(7)-C(6)-H(21)	109.2
C(6)-C(7)-C(8)	113.6	C(7)-C(8)-H(32)	120.3	C(6)-C(7)-C(8)	113.6
C(6)-C(7)-H(22)	109.2	C(9)-C(8)-H(32)	119.5	C(6)-C(7)-H(22)	109.2
C(6)-C(7)-H(23)	109.1	C(8)-C(9)-C(10)	121.5	C(6)-C(7)-H(23)	109.1
H(20)-C(6)-H(21)	106.1	C(8)-C(9)-H(33)	120.6	H(20)-C(6)-H(21)	106.1
C(8)-C(7)-H(22)	109.3	C(10)-C(9)-H(33)	117.9	C(8)-C(7)-H(22)	109.3
C(8)-C(7)-H(23)	109.3	C(9)-C(10)-C(11)	119.8	C(8)-C(7)-H(23)	109.3
C(7)-C(8)-C(9)	113.6	C(10)-C(11)-N(12)	119.3	C(7)-C(8)-C(9)	113.6
C(7)-C(8)-H(24)	109.3	C(10)-C(11)-H(34)	119.4	C(7)-C(8)-H(24)	109.3
C(7)-C(8)-H(25)	109.2	N(12)-C(11)-H(34)	121.4	C(7)-C(8)-H(25)	109.2
H(22)-C(7)-H(23)	106	C(11)-N(12)-N(13)	118.8	H(22)-C7-H(23)	106
C(9)-C(8)-H(24)	109.2	N(12)-N(13)-C(14)	129.9	C(9)-C(8)-H(24)	109.2
C(9)-C(8)-H(25)	109.2	N(12)-N(13)-H(35)	110.8	C(9)-C(8)-H(25)	109.2
C(8)-C(9)-C(10)	113.6	C(14)-N(13)-H(35)	119.3	C(8)-C(9)-C(10)	113.6

C(8)-C(9)-H(26)	109.2	N(13)-C(14)-O(15)	123.9	C(8)-C(9)-H(26)	109.2
C(8)-C(9)-H(27)	109.2	N(13)-C(14)-C(16)	113.3	C(8)-C(9)-H(27)	109.2
H(24)-C(8)-H(25)	106	O(15)-C(14)-C(16)	122.8	H(24)-C8-H(25)	106
C(10)-C(9)-H(26)	109.2	C(14)-C(16)-C(17)	110.9	C(10)-C(9)-H(26)	109.2
C(10)-C(9)-H(27)	109.2	C(14)-C(16)-H(36)	110.3	C(10)-C(9)-H(27)	109.2
C(9)-C(10)-C(11)	113.6	C(14)-C(16)-H(37)	107.9	C(9)-C(10)-C(11)	113.6
C(9)-C(10)-H(28)	109.2	C(17)-C(16)-H(36)	110.8	C(9)-C(10)-H(28)	109.2
C(9)-C(10)-H(29)	109.2	C(17)-C(16)-H(37)	109.4	C(9)-C(10)-H(29)	109.2
H(26)-C(9)-H(27)	106	C(16)-C(17)-C(18)	112.5	H(26)-C(9)-H(27)	106
C(11)-C(10)-H(28)	109.2	C(16)-C(17)-H(38)	109.1	C(11)-C10-H(28)	109.2
C(11)-C(10)-H(29)	109.2	C(16)-C(17)-H(39)	108.1	C(11)-C10-H(29)	109.2
C(10)-C(11)-C(12)	113.6	H(36)-C(16)-H(37)	107.5	C10-C(11)-C(12)	113.7
C(10)-C(11)-H(30)	109.2	C(18)-C(17)-H(38)	109.6	C10-C(11)-H((30)	109.2
C(10)-C(11)-H(31)	109.2	C(18)-C(17)-H(39)	110.5	C10-C(11)-H(31)	109.2
H(28)-C(10)-H(29)	106	C(17)-C(18)-C(19)	112.3	H(28)-C10-H(29)	106
C(12)-C(11)-H(30)	109.2	C(17)-C(18)-H(40)	109.7	C(12)-C(11)-H((30)	109.2
C(12)-C(11)-H(31)	109.2	C(17)-C(18)-H(41)	109.3	C(12)-C(11)-H(31)	109.2
C(11)-C(12)-C(13)	113.7	H(38)-C(17)-H(39)	106.9	C(11)-C(12)-C(13)	113.7
C(11)-C(12)-H(32)	109.3	C(19)-C(18)-H(40)	109.1	C(11)-C(12)-H((32)	109.3
C(11)-C(12)-H(33)	109.3	C(19)-C(18)-H(41)	109.1	C(11)-C(12)-H(33)	109.3
H(30)-C(11)-H(31)	106	C(18)-C(19)-C(20)	112.7	H((30))-C(11)-H(31)	106
C(13)-C(12)-H(32)	109.2	C(18)-C(19)-H(42)	109.2	C(13)-C(12)-H((32)	109.2
C(13)-C(12)-H(33)	109.2	C(18)-C(19)-H(43)	109.1	C(13)-C(12)-H(33)	109.2
C(12)-C(13)-C(14)	113.3	H(40)-C(18)-H(41)	107.2	C(12)-C(13)-C(14)	113.3
C(12)-C(13)-H(34)	109.2	C(20)-C(19)-H(42)	109.2	C(12)-C(13)-H(34)	109.2
C(12)-C(13)-H(35)	109.2	C(20)-C(19)-H(43)	109.3	C(12)-C(13)-H(35)	109.2
H(32)-C(12)-H(33)	106	C(19)-C(20)-C(21)	112.7	H((32)-C(12)-H(33)	106
C(14)-C(13)-H(34)	109.4	C(19)-C(20)-H(44)	109.2	C(14)-C(13)-H(34)	109.4
C(14)-C(13)-H(35)	109.4	C(19)-C(20)-H(45)	109.2	C(14)-C(13)-3H5	109.4
C(13)-C(14)-H(36)	111.2	H(42)-C(19)-H(43)	107.2	C(13)-C(14)-H(36)	111.2
C(13)-C(14)-H(37)	111.2	C(21)-C(20)-H(44)	109.2	C(13)-C(14)-H(37)	111.2
C(13)-C(14)-H(38)	111.5	C(21)-C(20)-H(45)	109.2	C(13)-C(14)-H(38)	111.5
H(34)-C(13)-H(35)	106	C(20)-C(21)-C(22)	112.7	H(34)-C(13)-H(35)	106
H(36)-C(14)-H(37)	107.5	C(20)-C(21)-H(46)	109.2	H(36)-C(14)-H(37)	107.5
H(36)-C(14)-H(38)	107.6	C(20)-(21)-H(47)	109.2	H(36)-C(14)-H(38)	107.6
H(37)-C(14)-H(38)	107.7	H(44)-C(20)-H(45)	107.2	H(37)-C(14)-H(38)	107.6
N(39)-C(40)-H(41)	122.1	C(22)-C(21)-H(46)	109.2	N(39)-C(40)-H(41)	121.4
N(39)-C(40)-C(42)	119.2	C(22)-C(21)-H(47)	109.2	N(39)-C(40)-C(42)	121.9
H(41)-C(40)-C(42)	118.7	C(21)-C(22)-C(23)	112.7	H(41)-C(40)-C(42)	116.6
C(40)-C(42)-C(43)	118.8	C(21)-C(22)-H(48)	109.2	C(40)-C(42)-C(43)	121.6
C(40)-C(42)-C(44)	124.8	C(21)-C(22)-H(49)	109.2	C(40)-C(42)-C(44)	119.4
C(43)-C(42)-C(44)	116.4	H(46)-C(21)-H(47)	107.2	C(43)-C(42)-C(44)	119
C(42)-C(43)-C(45)	121.8	C(23)-C(22)-H(48)	109.2	C(42)-C(43)-C(45)	120.2
C(42)-C(43)-H(46)	117.4	C(23)-C(22)-H(49)	109.2	C(42)C-(43)-H(46)	118.8
C(42)-C(44)-C(47)	122.1	C(22)-C(23)-C(24)	112.7	C(42)-C(44)-C(47)	120.6
C(42)-C(44)-N(52)	122.5	C(22)-C(23)-H(50)	109.2	C(42)-C(44)-H(48)	119.6
C(45)-C(43)-H(46)	120.8	C(22)-C(23)-H(51)	109.2	C(45)-C(43)-H(46)	121.1
C(43)-C(45)-C(48)	120.4	H(48)-C(22)-H(49)	107.2	C(43)-C(45)-C(49)	120.4
C(43)-C(45)-H(49)	119.5	C(24)-C(23)-H(50)	109.2	C(43)-C(45)-H((50)	119.7
C(47)-C(44)-N(52)	115.4	C(24)-C(23)-H(51)	109.2	C(47)-C(44)-H(48)	119.8
C(44)-C(47)-C(48)	119.9	C(23)-C(24)-C(25)	112.8	C(44)-C(47)-C(49)	120

C(44)-C(47)-H(50)	118.5	C(23)-C(24)-H(52)	109.2	C(44)-C(47)-H(51)	119.9
C(44)-N(52)-O(53)	117.4	C(23)-C(24)-H(53)	109.2	C(49)-C(45)-H((50)	119.9
C(44)-N(52)-O(54)	118.4	H(50)-C(23)H-(51)	107.2	C(45)-C(49)-C(47)	119.8
C(48)-C(45)-H(49)	120.1	C(25)-C(24)-H(52)	109.1	C(45)-C(49)-H(52)	120.1
C(45)-C(48)-C(47)	119.4	C(25)-C(24)-H(53)	109.1	C(49)-C(47)-H(51)	120.2
C(45)-C(48)-H(51)	120.7	C(24)-C(25)-C(26)	112.3	C(47)-C(49)-H(52)	120.1
C(48)-C(47)-H(50)	121.6	C(24)-C(25)-H(54)	109.1		
C(47)-C(48)-H(51)	119.9	C(24)-C(25)H-(55)	109.1		
O(53)-N(52)-O(54)	124.1	H(52)-C(24)-H(53)	107.1		
		C(26)-C(25)-H(54)	109.6		
		C(26)-C(25)-H(55)	109.6		
		C(25)-C(26)-H(56)	110.5		
		C(25)-C(26)-H(57)	110.5		
		C(25)-C(26)-H(58)	111.2		
		H(54)-C(25)-H(55)	107.1		
		H(56)-C(26)-H(57)	108.1		
		H(56)-C(26)-H(58)	108.2		
		H(57)-C(26)-H(58)	108.2		

Table S3: Natural bond orbital (NBO) analysis of NBDH, NMDH and FBDH

Molecules	Donor (i)	Type	Acceptor (j)	Type	E(2)[a]	E(j)–E(i)[b] [a.u.]	F(i; j)[c] [a.u.]
	C 4 - H17	σ	C2 - O3	π*	6.34	0.53	0.054
	N39 - C40	π	C42 - C44	π*	10.84	0.35	0.06
	C42 - C43	σ	C44 - N52	σ*	5.52	0.96	0.066
	C42 - C44	σ	C44 - C47	σ*	5.05	1.27	0.072
	C42 - C44	π	N39 - C40	π*	13.58	0.28	0.058
	C42 - C44	π	C43 - C45	π*	15.93	0.3	0.064
	C42 - C44	π	C47 - C48	π*	20.22	0.3	0.071
	C42 - C 44	π	N52 - O53	π*	23.69	0.15	0.056
	C43 - C45	π	C42 - C44	π*	21.68	0.27	0.07
	C43 - C45	π	C47 - C48	π*	18.99	0.28	0.066
	C44 - C47	σ	C42 - C44	σ*	5.6	1.26	0.075
**NBDH**	C47 - C48	π	C42 - C44	π*	19.86	0.28	0.068
	C47 - C48	π	C43 - C45	π*	19.29	0.29	0.068
	C47 - H50	σ	C42 - C44	σ*	5.37	1.06	0.068
	N52 - O53	π	N52 - O53	π*	7.14	0.33	0.052
	N1	LP(1)	C2 - O3	π*	46.24	0.31	0.11
	N1	LP(1)	N39 - C40	π*	30.98	0.28	0.087
	O3	LP(2)	N1 - C2	σ*	29.41	0.66	0.127
	O3	LP(2)	C2 - C4	σ*	19.92	0.62	0.102
	N39	LP(1)	N1 - H15	σ*	8.37	0.76	0.072
	N39	LP(1)	C40 - H41	σ*	8.86	0.8	0.076
	O53	LP (2)	C44 - N52	σ*	13.73	0.56	0.078

	O53	LP (2)	N52 - O54	σ*	18.94	0.71	0.104
	O54	LP (2)	C44 - N52	σ*	12.28	0.56	0.074
	O54	LP (2)	N52 - O53	σ*	18.71	0.72	0.105
	O54	LP (3)	N52 - O53	π*	160.86	0.15	0.143
	O54	LP (3)	N52 - O54	σ*	7.77	0.7	0.075
	C1-C6	π	C2-C3	π*	16.63	0.28	0.064
	C1-C6	π	C4-C5	π*	16.62	0.28	0.064
	C1-C6	π	C7-C8	π*	17.18	0.27	0.065
	C1-C6	π	C9-C10	π*	16.47	0.28	0.063
	C2-C3	π	C1-C6	π*	16.42	0.29	0.065
	C2-C3	π	C4-C5	π*	17.43	0.3	0.064
	C4-C5	π	C1-C6	π*	16.35	0.29	0.065
	C4-C5	π	C2-C3	π*	17.84	0.3	0.065
	C7-C8	π	C1-C6	π*	16.67	0.29	0.065
	C7-C8	π	C9-C10	π*	19.09	0.3	0.068
	C9-C10	π	C1-C6	π*	17.09	0.29	0.065
**NMDH**	C9-C10	π	C7-C8	π*	17.66	0.29	0.065
	C9-C10	π	C11 - N12	π*	16.36	0.27	0.061
	C10-C12	σ	N12-N13	σ*	5.24	1.02	0.065
	C11-N12	π	C9 - C10	π*	7.57	0.37	0.051
	C14-C16	σ	N12-N13	σ*	5.26	1	0.065
	C16-H37	σ	C14-O15	π*	6.06	0.52	0.053
	N12	LP (1)	C11 - H34	σ*	8.55	0.83	0.076
	N12	LP (1)	N13 - C14	σ*	11.4	0.82	0.087
	N13	LP (1)	C11 - N12	π*	27.34	0.29	0.083
	N13	LP (1)	C14 - O15	π*	56.87	0.29	0.117
	O15	LP (2)	N13 - C14	σ*	25.24	0.7	0.12
	O15	LP (2)	C14 - C16	σ*	18.8	0.63	0.099
	C4-H17	π	C2 - O3	π*	6	0.54	0.053
	C42-C44	π	N39-C40	π*	18.96	0.26	0.066
	C42-C44	π	C43-C45	π*	18.63	0.29	0.067
	C42-C44	π	C47-C49	π*	19.99	0.28	0.068
	C43-C45	π	C42-C44	π*	20.1	0.28	0.068
	C43-C45	π	C47-C49	π*	20.94	0.28	0.069
	C47-C49	π	C42-C44	π*	20.58	0.28	0.069
**FBDH**	C47-C49	π	C43-C45	π*	18.75	0.29	0.067
	N 1	LP (1)	C2-O3	π*	47.17	0.31	0.111
	N1	LP(1)	N39-C40	π*	29.03	0.29	0.084
	O3	LP(2)	N1-C2	σ*	28.81	0.67	0.126
	O3	LP(2)	C2-C4	σ*	20.05	0.62	0.102
	N39	LP(1)	C40-C41	σ*	10.13	0.76	0.079
	C 49 - F52	σ	C 43 - C45	σ*	1.35	1.75	0.043
	C49 - F 52	σ	C 44 - C47	σ*	1.4	1.73	0.044

[a]$E^{(2)}$ means energy of hyper conjugative interaction (stabilization energy in kcal/mol).
[b]Energy difference between donor and acceptor i and j NBO orbitals.
[c]F(i; j) is the Fock matrix element between i and j NBO orbitals

Table S4: Vibrational frequencies of NBDH with proposed assignments

frequency (cm^{-1}) (Theo) Unscaled	frequency (cm^{-1}) (Theo) Scaled	I_{IR}	Vibrational assignments
3489.05	3370.42	4.1626	υ N-H_{DH}
3224.67	3115.03	1.7179	υ (s) C–H_{Ben}
3208.35	3099.27	3.3055	υ (s) C–H_{Ben}
3193.76	3085.17	9.4475	υ (as) C–H_{Ben}+ υ (s) C–H_{Ben}
3177.47	3069.44	3.2634	υ (as) H–C=C–H_{Ben}
3127.52	3021.18	30.9424	υ C–H_{DH}
3084.10	2979.24	48.1115	υ (as) C–H_{Meth}
3082.00	2977.21	48.8813	υ (as) C–H_{Eth}
3079.35	2974.65	77.1880	υ (as) C–H_{Eth} + υ (as) C–H_{Meth}
3041.59	2938.18	14.3644	υ (as) C–H_{Eth} + υ (s) C–H_{Eth}
908.23	877.35	3.1942	υ (τ) C–H_{Ben}
908.23	877.35	0.3660	υ (w) C–H_{Ben}
881.28	851.32	9.5792	υ (τ) C–H_{Eth}
853.63	824.61	19.0694	υ (δ) N=O + υ (s) C=C–C=C–C_{Ben}
826.85	798.74	0.3875	υ (ρ) C–H_{Eth}
762.53	736.60	30.6766	υ (w) C–H_{Ben}
708.11	684.03	17.9713	υ (δ) C–H_{Ben} + υ (δ) N=O + υ (s) C–C=C–C_{Ben}
649.87	627.77	7.1000	υ (s) C=C–C=C–C_{Ben}

υ, stretching; δ, scissoring; ρ, rocking; w, wagging; s, symmetric; as, asymmetric; τ, twisting; Ben, benzene ring; Meth, methyl group; DH, Dodecanehydrazide; Eth, ethyl group; Exp, experimental; Theo, theoretical

Table S5: Vibrational frequencies of NMDH with proposed assignments

frequency (cm^{-1}) (Theo) Unscaled	frequency (cm^{-1}) (Theo) Scaled	I_{IR}	Vibrational assignments
3486.67	3368.12	8.0552	υ N–H_{DH}
3199.97	3091.17	2.1249	υ (s) C–H_{Ben}
3198.75	3089.99	19.3163	υ (s) C–H_{Ben}
3184.94	3076.65	37.4448	υ (as) C–H_{Ben}
3163.01	3055.47	1.5980	υ (as) C–H_{Ben}
3083.93	2979.08	48.3591	υ (as) C–H_{Meth}
3081.83	2977.05	49.0545	υ (as) C–H_{Eth}
3049.85	2946.16	41.4160	υ C–H_{DH}

3024.17	2921.35	36.1823	υ (s) C–H$_{Eth}$
3018.39	2915.76	53.5408	υ (s) C–H$_{Meth}$
732.61	707.70	4.9204	υ (ρ) C–H$_{Ben}$
661.65	639.15	14.8127	υ (as) C–C=C–C=C–C=C–C=C–C$_{Ben}$
654.61	632.35	0.1614	υ (τ) C–H$_{Ben}$ + υ (as) C–C=C–C=C–C=C–C=C–C$_{Ben}$
622.50	601.34	4.0741	υ (ρ) C–H$_{Eth}$
562.42	543.30	1.6392	υ (w) C–H$_{Ben}$ + υ (s) C–C=C–C=C–C=C–C=C–C$_{Ben}$
546.69	528.10	3.6126	υ (as) C–C=C–C=C–C=C–C=C–C$_{Ben}$

υ, stretching; δ, scissoring; ρ, rocking; *w*, wagging; s, symmetric; as, asymmetric; τ, twisting; Ben, benzene ring; Meth, methyl group; DH, Dodecanehydrazide; Eth, ethyl group; Exp, experimental; Theo, theoretical

Table S6: Vibrational frequencies of FBDH with proposed assignments

frequency (cm^{-1}) (Theo) Unscaled	frequency (cm^{-1}) (Theo) Scaled	I_{IR}	Vibrational assignments
3491.28	3372.58	7.1366	υ N-H$_{DH}$
3205.95	3096.95	3.4373	υ (s) C–H $_{Ben}$
3201.42	3092.57	5.1257	υ C–H $_{Ben}$
3193.01	3084.45	1.7980	υ (as) C–H $_{Ben}$
3084.01	2979.15	48.1386	υ (as) C–H$_{Meth}$
3081.85	2977.07	48.9623	υ (as) C–H$_{Eth}$
3079.30	2974.60	77.2423	υ (as) C–H$_{Eth}$ + υ (as) C–H$_{Meth}$
3055.12	2951.25	205.8827	υ (as) C–H$_{Eth}$
3041.88	2938.46	17.3727	υ (s) C–H$_{Eth}$ + υ (as) C–H$_{Eth}$
1028.15	993.19	2.5078	υ (s) C=C–C=C–C$_{Ben}$
942.11	910.08	4.8607	υ (τ) C–H$_{Ben}$
938.42	906.51	0.8676	υ (τ) C–H$_{Eth}$
896.77	866.28	1.5364	υ (w) C–H$_{Meth}$
880.68	850.74	3.0374	υ (δ) C–H$_{Eth}$
827.11	798.99	6.9598	υ (w) C–H$_{Ben}$
804.57	777.21	17.0773	υ (s) C=C–C=C–C$_{Ben}$ + υ C–F$_{Ben}$
732.25	707.35	5.2830	υ (ρ) C–H$_{Eth}$
651.23	629.09	2.7356	υ (s) C=C–C=C–C$_{Ben}$
634.17	612.61	16.6578	υ (ρ) C–H$_{Eth}$

υ, stretching; δ, scissoring; ρ, rocking; *w*, wagging; s, symmetric; as, asymmetric; τ, twisting; Ben, benzene ring; Meth, methyl group; DH, Dodecanehydrazide; Eth, ethyl group; Exp, experimental; Theo, theoretical

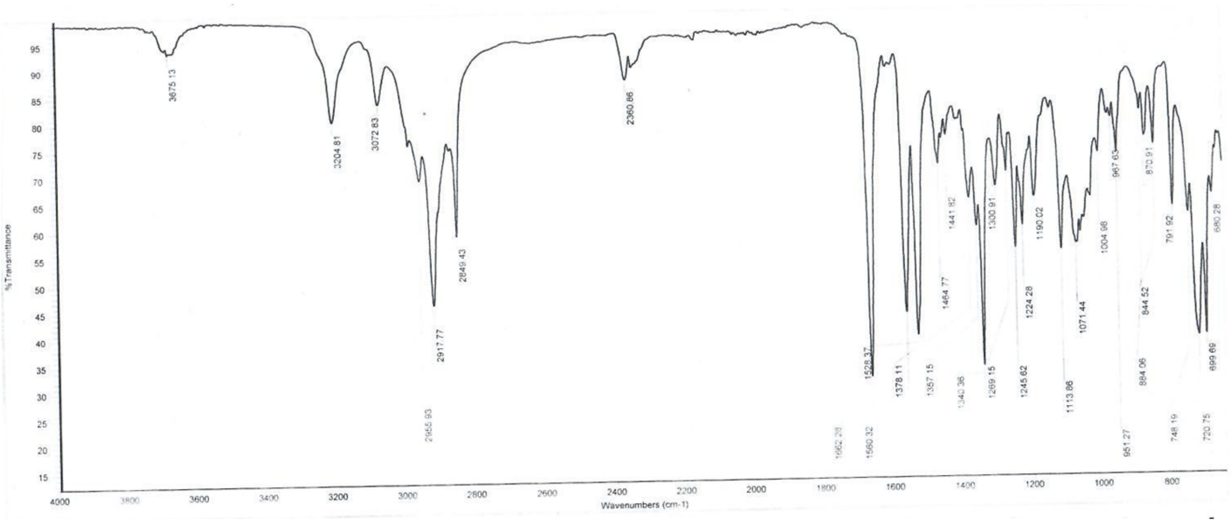

Figure S1: Experimental FTIR spectrum of **NBDH**

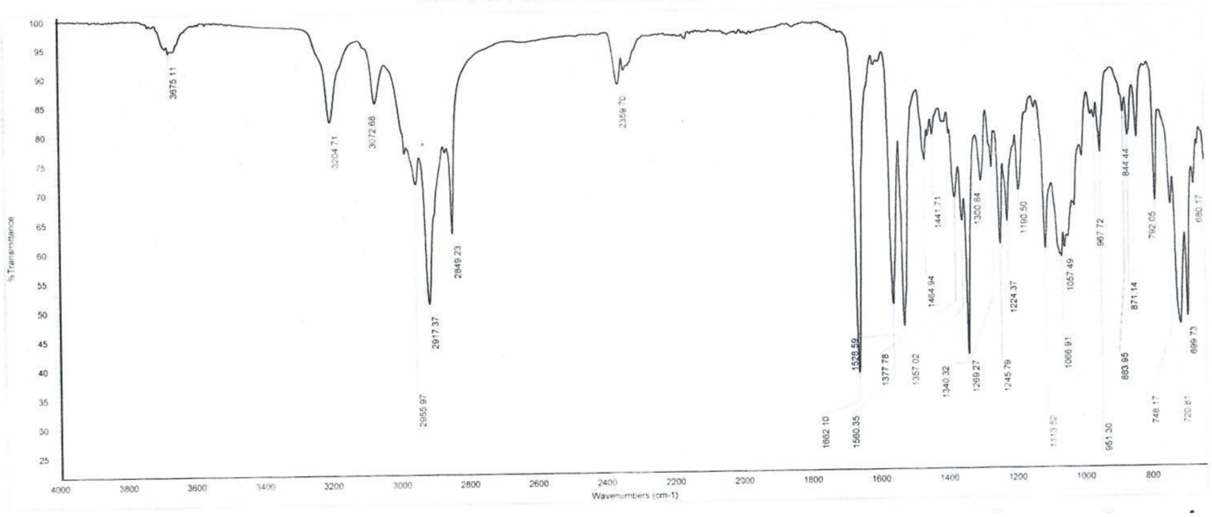

Figure S2: Experimental FTIR spectrum of **NMDH**

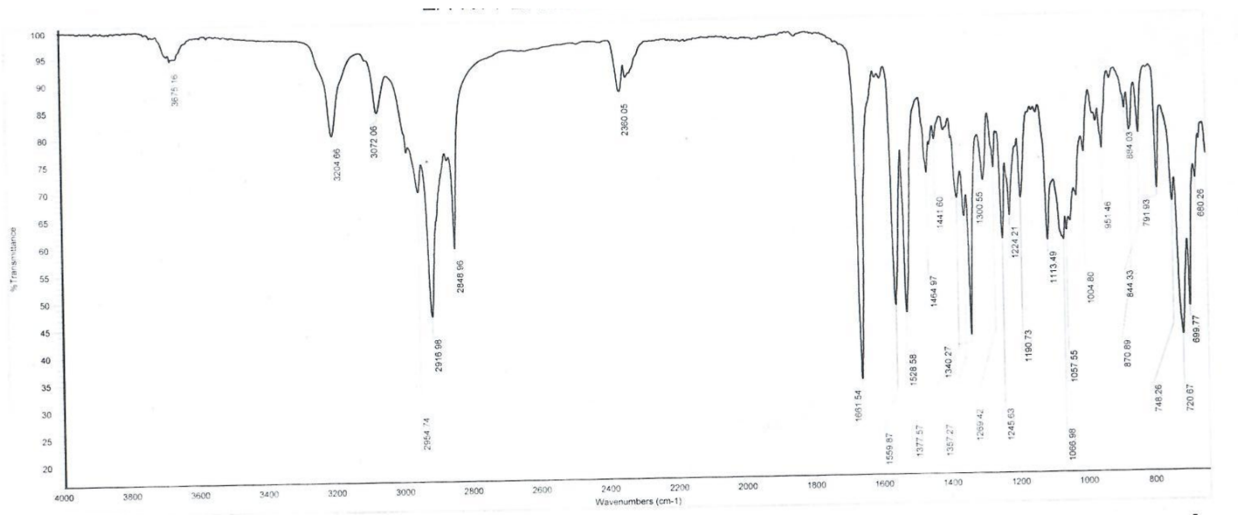

Figure S3: Experimental FTIR spectrum of **FBDH**

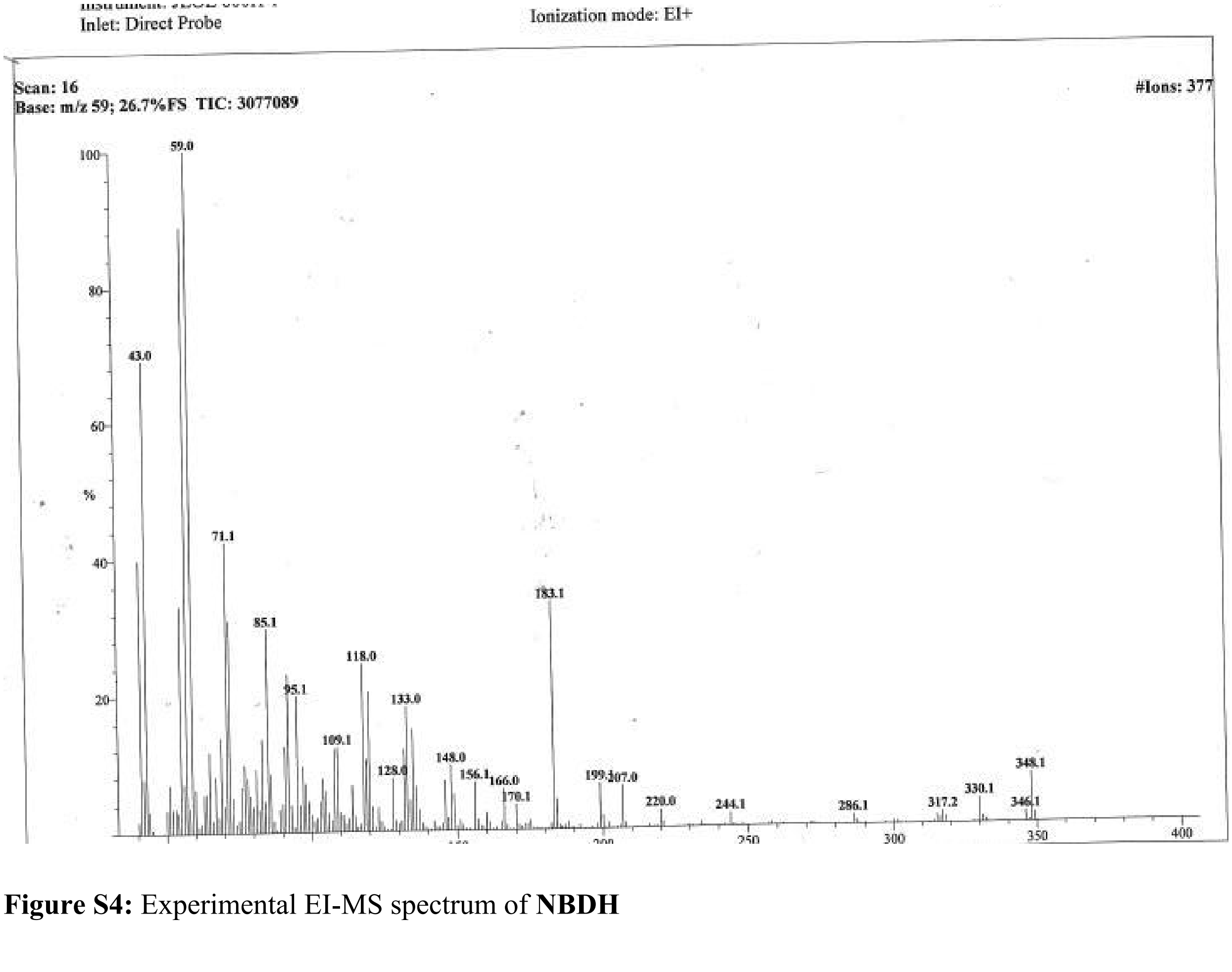

Figure S4: Experimental EI-MS spectrum of **NBDH**

Inlet: Direct Probe Ionization mode: EI+

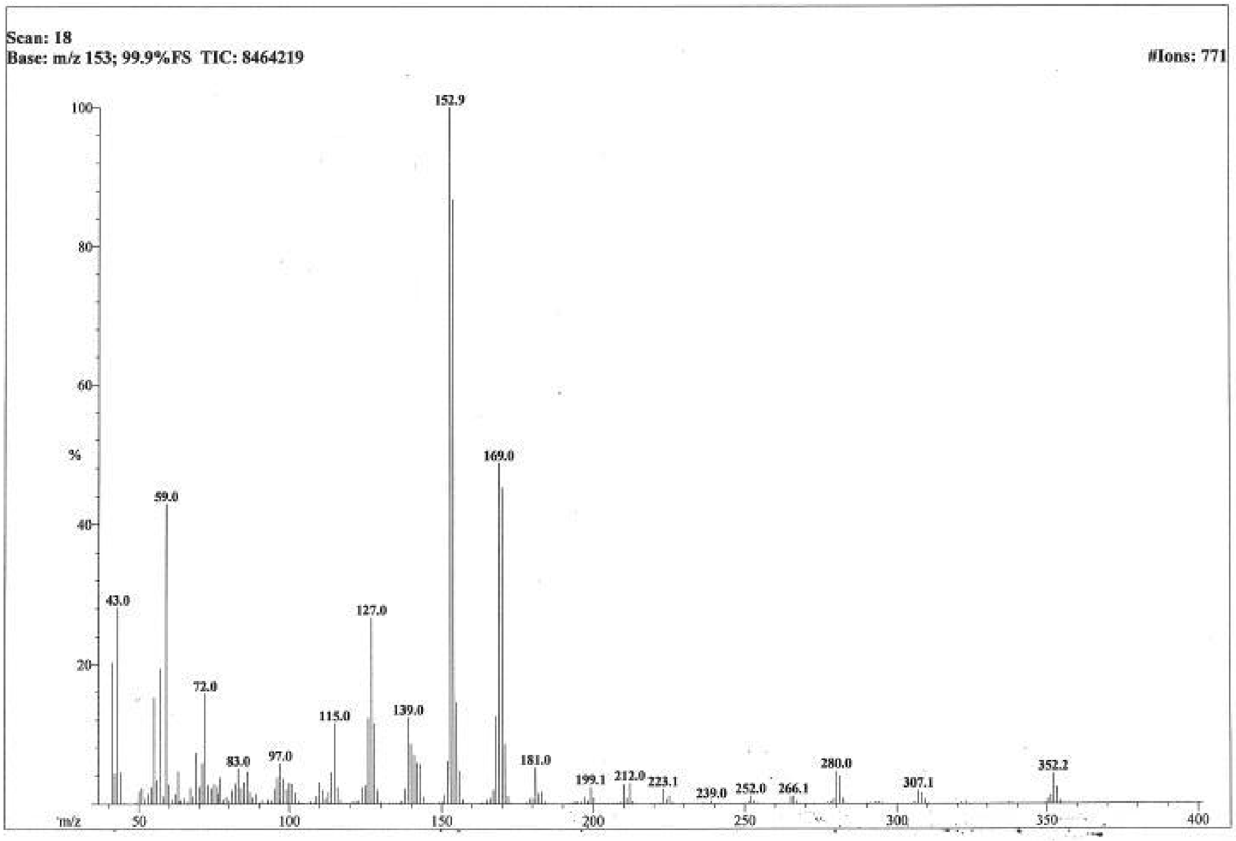

Figure S5: Experimental EI-MS spectrum of **NMDH**

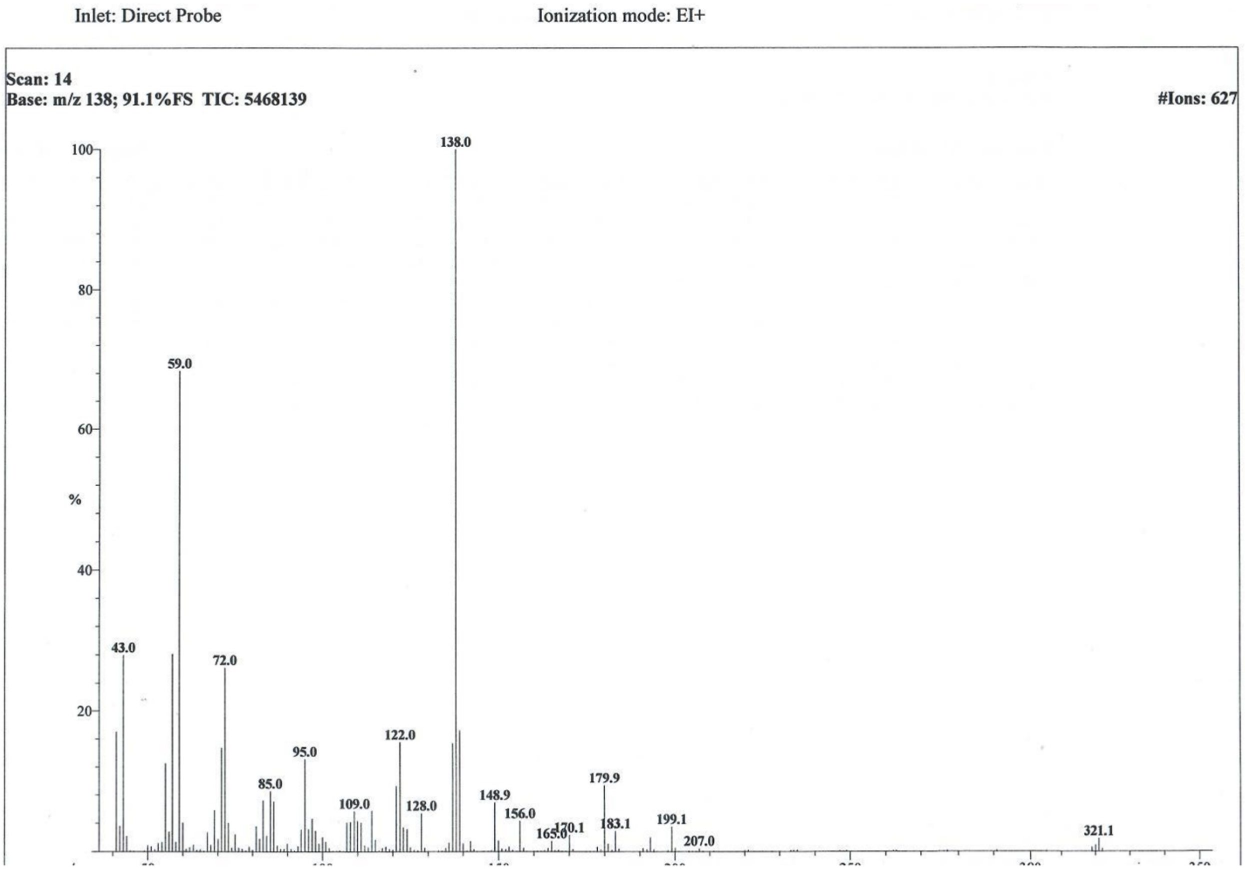

Figure S6: Experimental EI-MS spectrum of **FBDH**

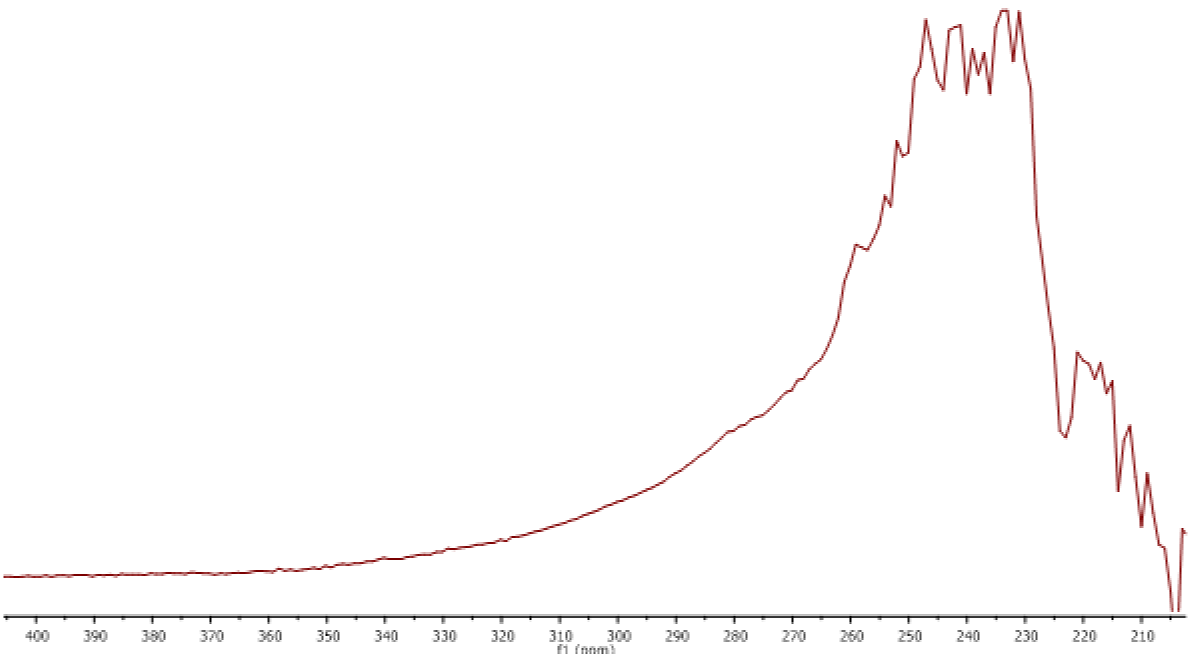

Figure S7: Experimental UV spectrum of the compound **NBDH**

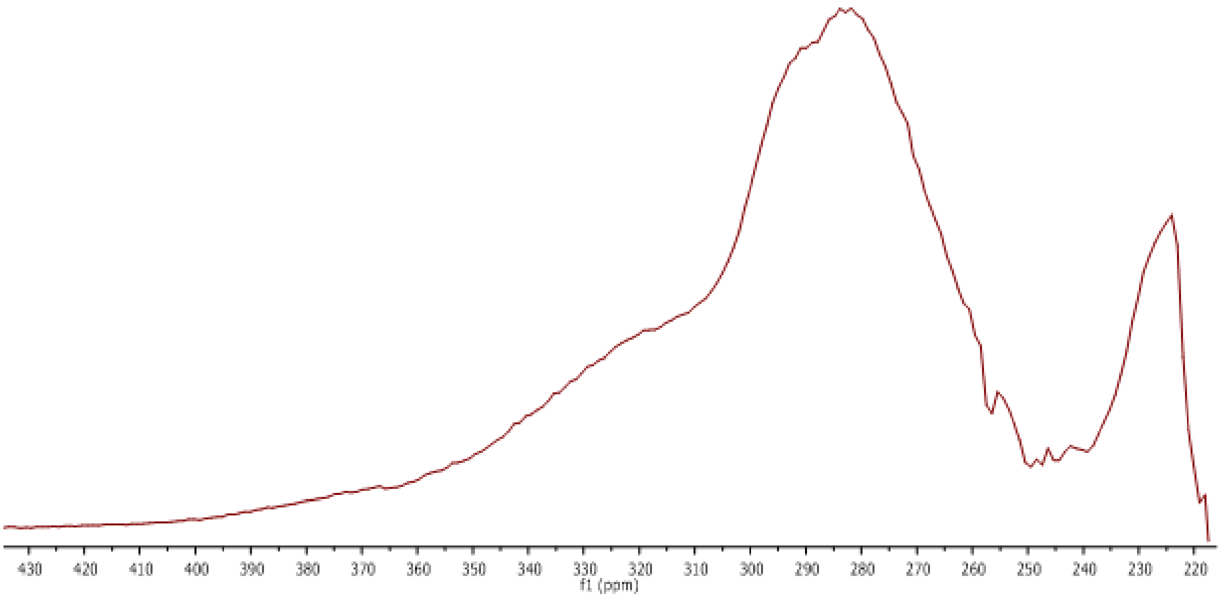

Figure S8: Experimental UV spectrum of the compound **NMDH**

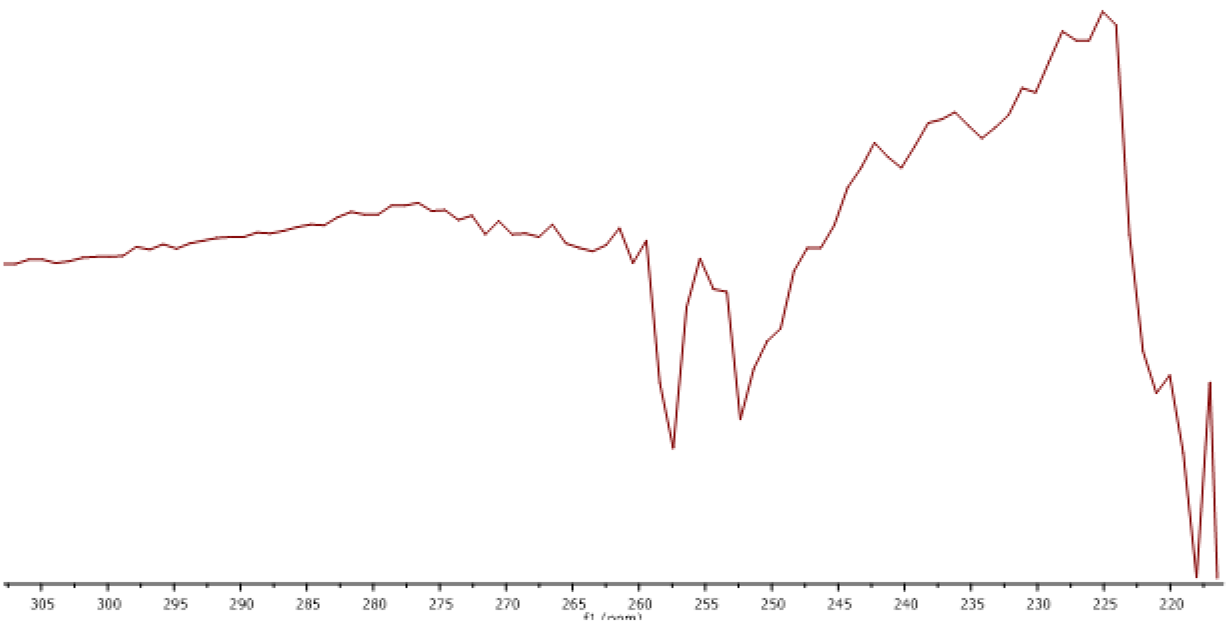

Figure S9: Experimental UV spectrum of the compound **FBDH**

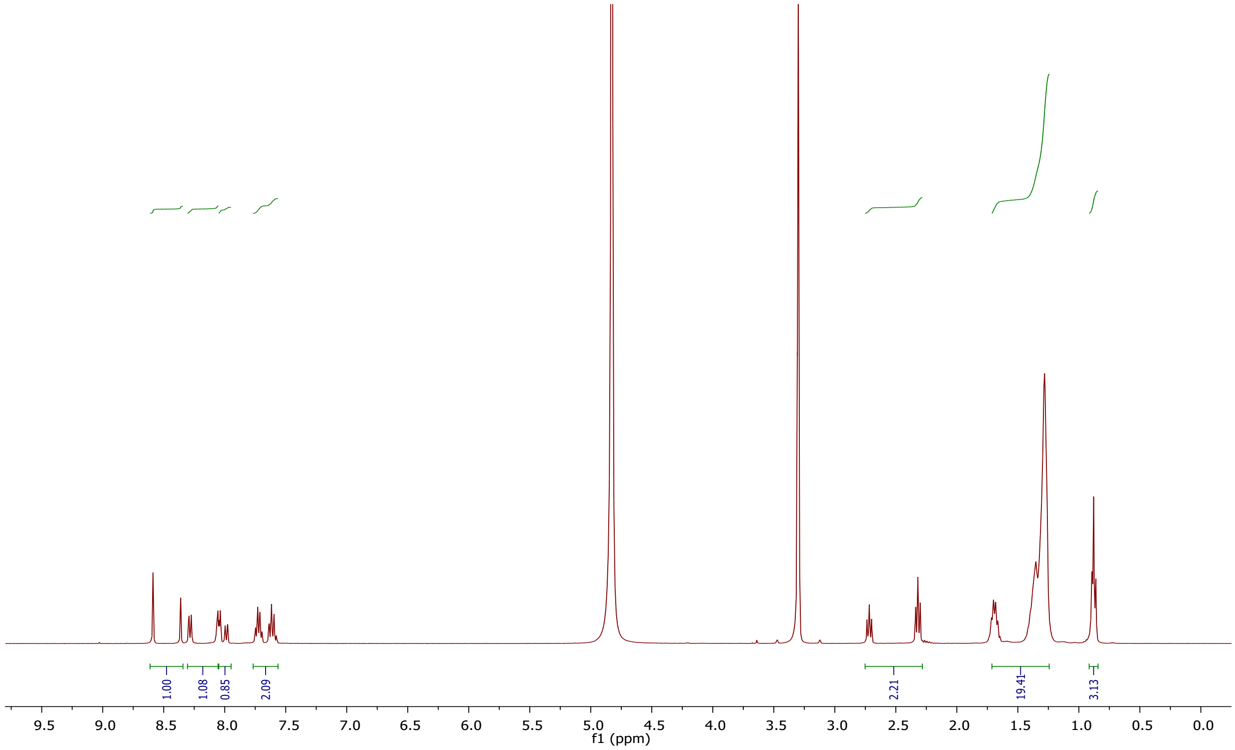

Figure S10: ^{1}H spectrum of (*E*)-N'-(2-nitrobenzylidene)dodecanehydrazide (NBDH).

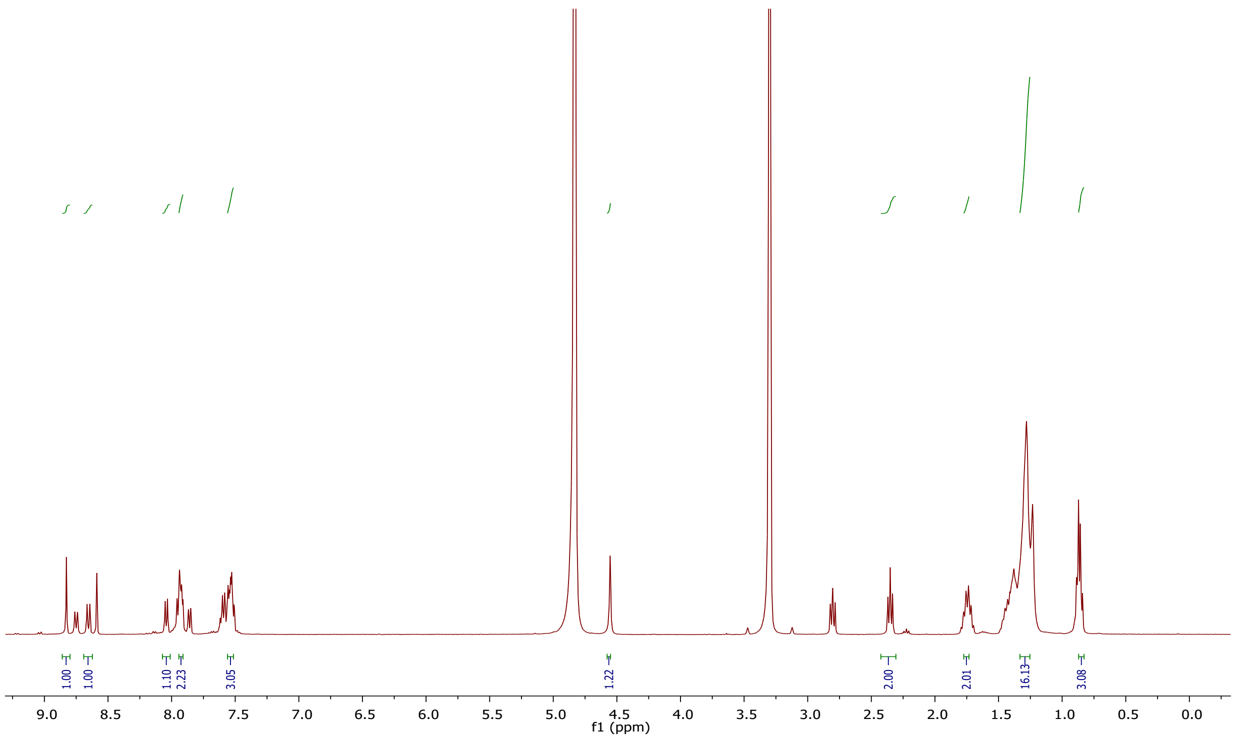

Figure S11:^{1}H spectrum of (*E*)-N'-(naphthalen-1-ylmethylene)dodecanehydrazide (NMDH).

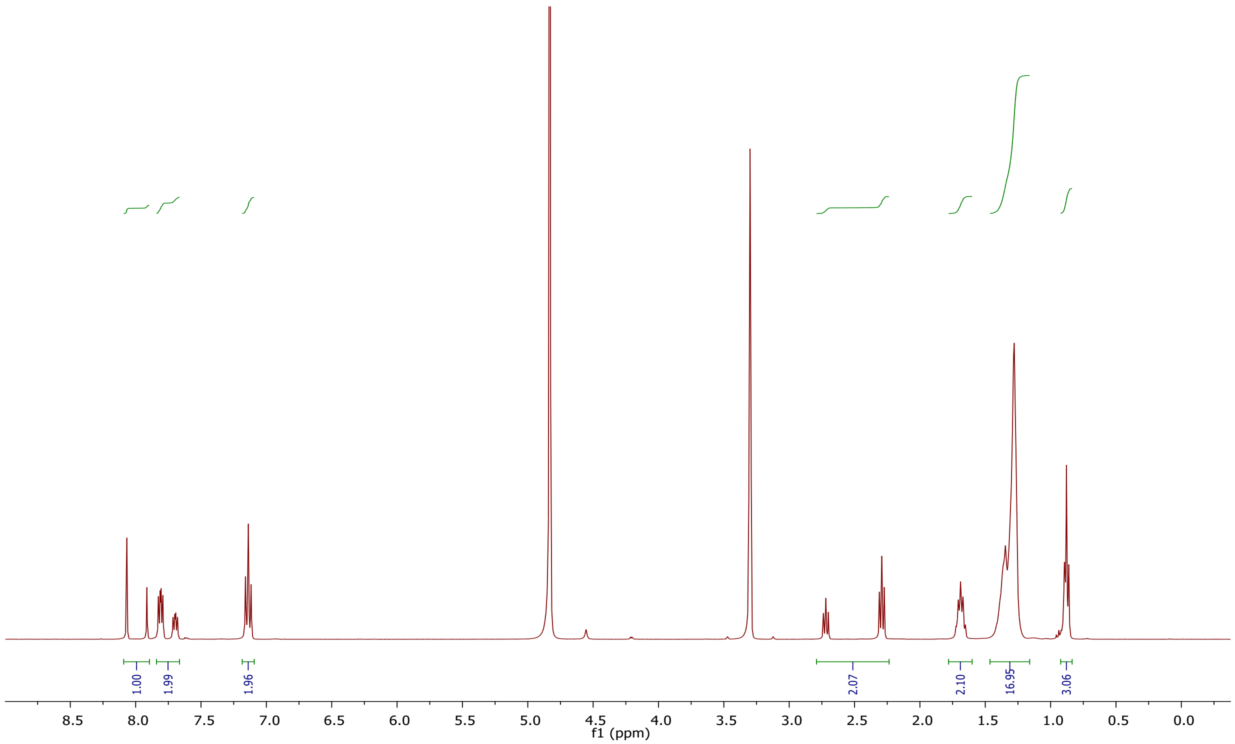

Figure S12:^{1}H spectrum of (*E*)-N'-(4-fluorobenzylidene)dodecanehydrazide (FBDH).

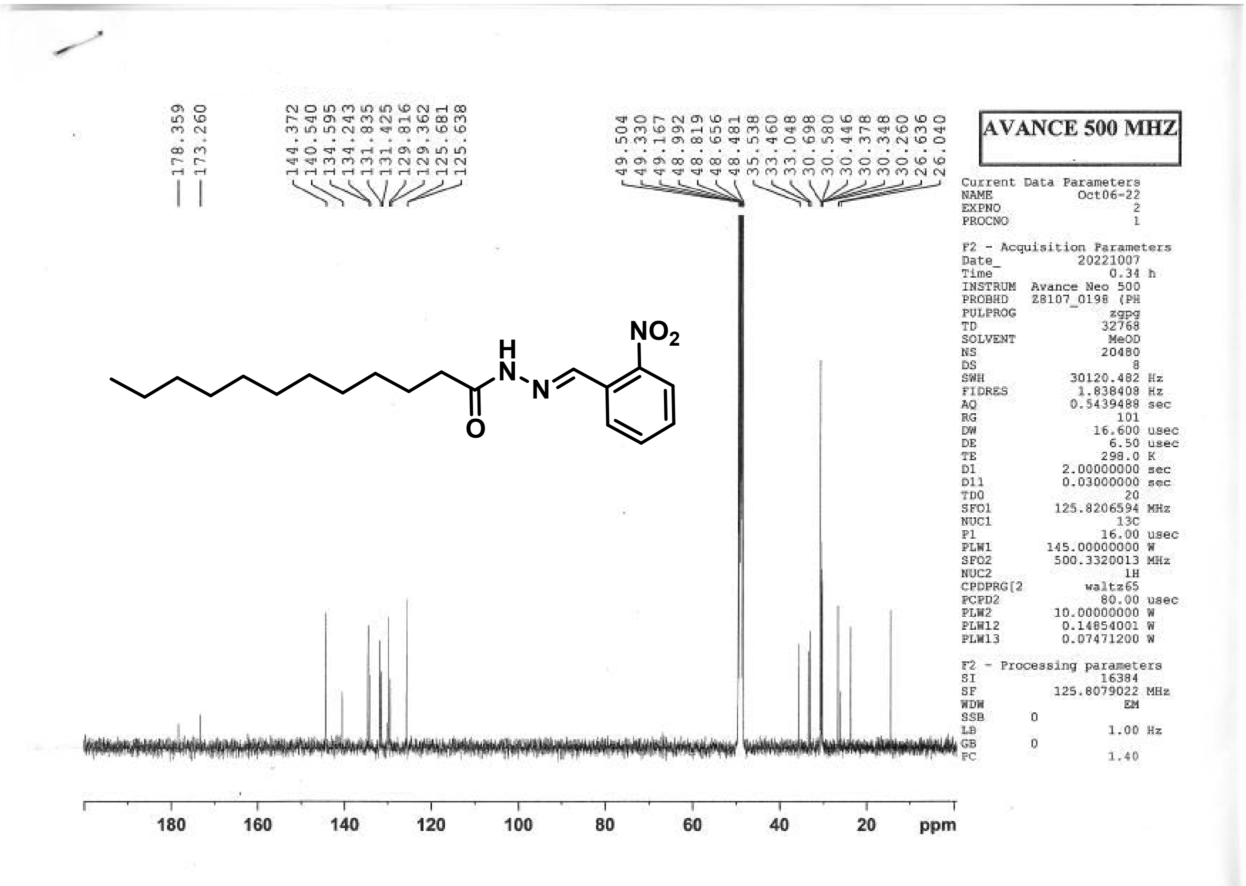

Figure S13:C-NMR spectrum of NBDH.

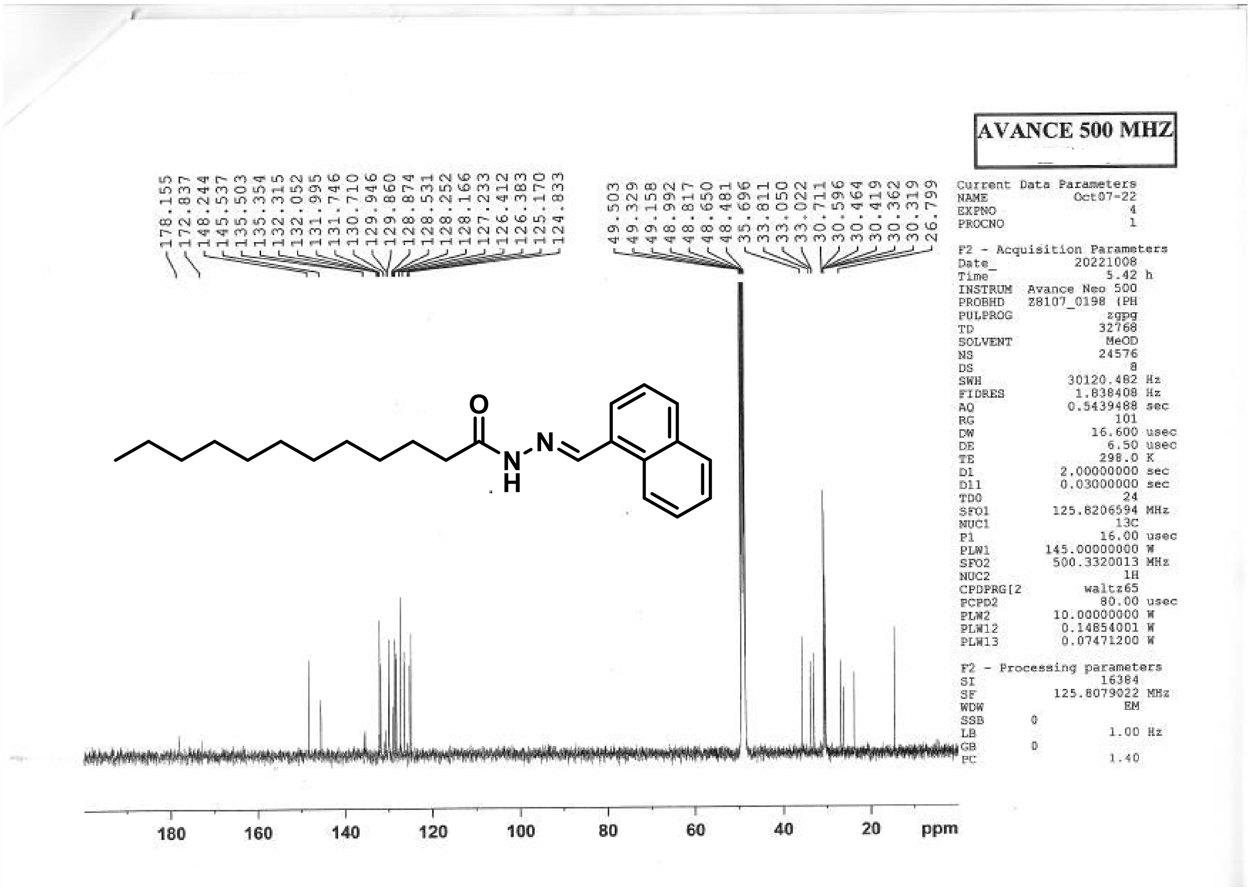

Figure S14:C-NMR spectrum of NMDH.

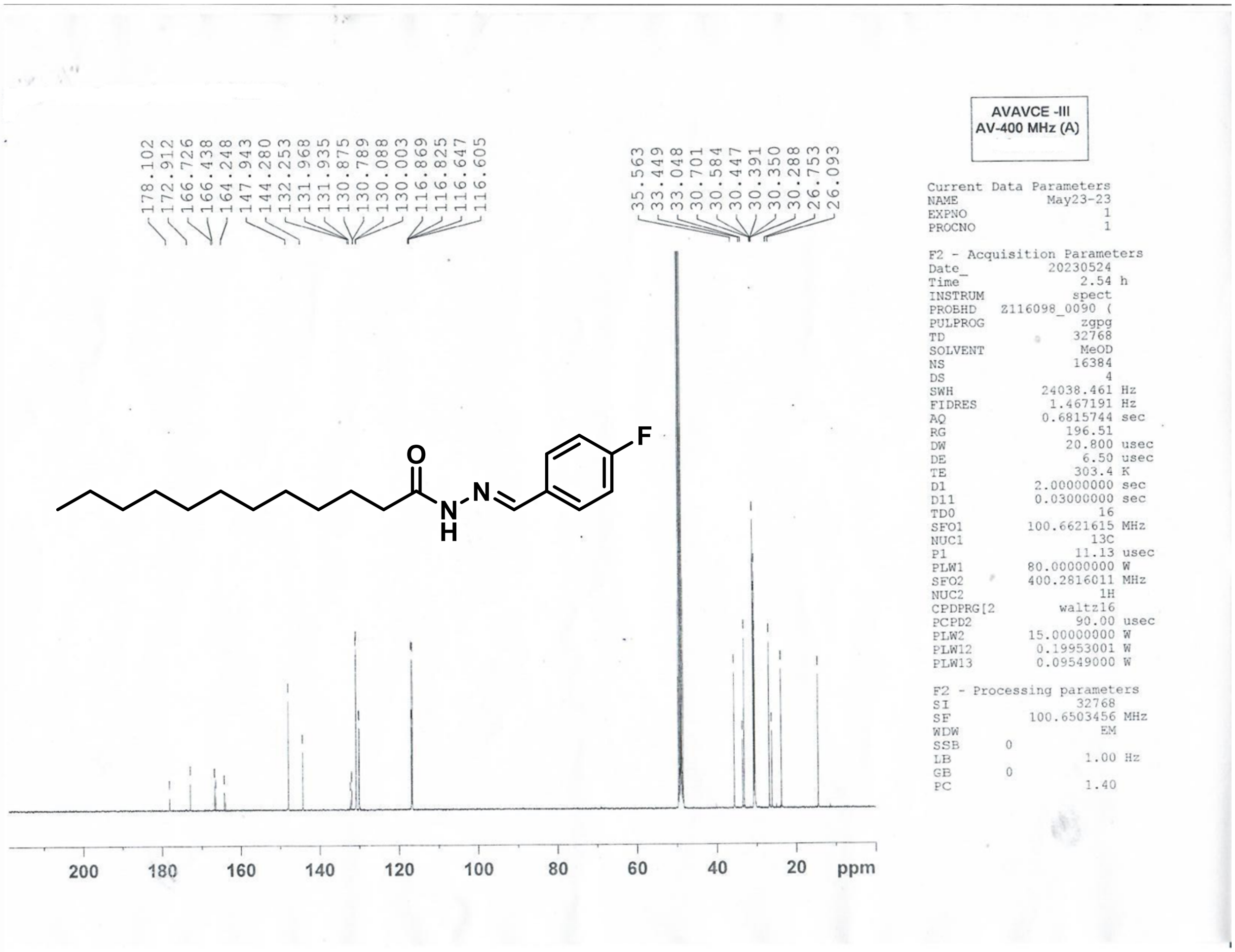

Figure S15:C-NMR spectrum of FBDH.
